# Exercise Modulates *miR-276a/nej* Expression and Function in the *Drosophila* Heart: A Possible Contribution of the Fat Body

**DOI:** 10.3390/ijms27156640

**Published:** 2026-07-25

**Authors:** Qin Yi, Chao Tang, Qiufang Li, Meng Ding, Zhihao Pan, Zhengwen Yu, Xu Ping, Wenzhi Gu, Yuepeng Li, Ni Ding, Wenqing Huang, Jin Dai, Xinrui Xu, Shiyi He, Lan Zheng

**Affiliations:** Key Laboratory of Physical Fitness and Exercise Rehabilitation of Hunan Province, Hunan Normal University, Changsha 410012, China

**Keywords:** cardiac function, *nej*, *miR-276a*, exercise, fat body

## Abstract

Cardiac rhythm and pumping function are essential outputs of cardiac homeostasis and depend on precise molecular regulation. The histone acetyltransferases CBP/p300 have been implicated in cardiac remodeling and functional regulation. nej, the *Drosophila* homolog of CBP/p300, may therefore represent an important regulator of cardiac function. However, the relationship between *miR-276a* and nej in the heart, as well as the potential involvement of the fat body in this regulatory relationship, remains unclear. Using *Drosophila* as a model, we first examined the effects of cardiac-specific nej overexpression and knockdown on cardiac function and tested the interaction between *miR-276a* and the nej 3′ untranslated region using a dual-luciferase reporter assay. We subsequently manipulated *miR-276a* using Hand-Gal4 and Cg-Gal4 and assessed *miR-276a* and *nej* expression in cardiac samples, cardiac function, and climbing ability. Finally, we investigated the effects of an exercise intervention on cardiac *miR-276a*/*nej* expression and functional outcomes. Both cardiac-specific overexpression and knockdown of *nej* impaired cardiac rhythm, reduced pumping function, and decreased climbing ability. The reporter assay supported a functional interaction between *miR-276a* and the *nej* 3′ untranslated region, while cardiac-specific *miR-276a* manipulation was accompanied by inverse changes in *nej* mRNA expression and by cardiac and climbing abnormalities. Cg-Gal4-mediated *miR-276a* upregulation or downregulation was associated with altered *miR-276a* and *nej* expression in cardiac samples and with cardiac and climbing impairments. Exercise improved cardiac and climbing ability in both cardiac *miR-276a* knockdown and overexpression groups. It increased cardiac *miR-276a* expression and decreased *nej* expression in the cardiac knockdown group but did not significantly alter either transcript in the cardiac overexpression group. Exercise also improved cardiac function in both Cg-Gal4-mediated *miR-276a* knockdown and overexpression groups. In the Cg-Gal4-mediated knockdown group, this improvement was accompanied by increased *miR-276a* and decreased *nej* expression in cardiac samples. These findings indicate that exercise modulates cardiac function and *miR-276a*/*nej* expression in a context-dependent manner and suggests a possible contribution of the fat body.

## 1. Introduction

Cardiac rhythm and pumping function are core outputs of cardiac homeostasis [1,2]. Their stability depends on coordinated regulation of myocardial structure, contractile performance, and molecular signaling [3]. Disruption of these processes can lead to arrhythmia and impaired pumping function, which are major pathological features of cardiac dysfunction [4]. Therefore, identifying the molecular mechanisms that maintain cardiac rhythm and pumping capacity is important for understanding cardiac functional regulation.

The histone acetyltransferase CBP/p300 is one of the key molecules involved in regulating cardiac function [5]. It contributes to the development of cardiac arrhythmias and cardiac pumping dysfunction by regulating the expression of genes associated with myocardial fibrosis and hypertrophy [6,7]. For example, studies have shown that CBP/p300 influences cardiac rhythm function by participating in the regulation of myocardial fibrosis through various mechanisms [8]. In mice, cardiac p300 overexpression has been shown to induce cardiac hypertrophy and functional impairment [9]. In *Drosophila*, the CBP/p300 homolog is *nej* [10]. These findings suggest that abnormal *nej* expression may disrupt cardiac function in *Drosophila*; however, the upstream mechanisms that regulate cardiac *nej* expression remain unclear.

Post-transcriptional regulation is a key layer of gene expression control. MiRNAs have emerged as important regulators of cardiac development, remodeling and function by targeting *mRNAs* [11,12].It is therefore important to identify miRNAs that regulate *nej* in the heart. In C4da neurons of the *Drosophila*, *miR-276a* regulates dendritic development by inhibiting the expression of *nej* [13]. However, whether *miR-276a* also targets *nej* in the heart and affects cardiac function remains unclear.

In addition to cardiac regulation, *miR-276a* manipulation in other tissues may be associated with changes in cardiac function. The *Drosophila* fat body, which performs metabolic and endocrine functions analogous in part to those of mammalian adipose tissue, stores energy and releases bioactive factors involved in systemic metabolic regulation [14,15]. Adipose tissue-derived miRNAs can participate in and regulate gene expression in distant tissues [15,16], and emerging evidence suggests that this mode of regulation may contribute to cardiac function [16,17,18]. To explore the possible involvement of the fat body, we used Cg-Gal4, a driver commonly employed for gene manipulation in the *Drosophila* fat body, and examined whether Cg-Gal4-mediated *miR-276a* manipulation was associated with changes in *miR-276a* and *nej* expression in cardiac samples and with cardiac functional phenotypes.

Exercise is an effective cardioprotective intervention that can prevent or improve cardiac arrhythmia and pumping dysfunction [19,20,21]. Its protective effects have also been associated with changes in miRNA expression and acetylation-related regulation [20,22]. However, whether exercise affects cardiac *miR-276a*/*nej* expression and whether these molecular changes are consistently associated with functional improvement remains unclear. The possible contribution of the fat body to these exercise-related responses also requires further investigation.

Consequently, using *Drosophila* as a model organism, this study aimed to: (1) determine the effects of cardiac-specific *nej* manipulation on cardiac function and climbing ability; (2) examine the interaction between *miR-276a* and the *nej* 3′ untranslated region and assess the effects of cardiac *miR-276a* manipulation on cardiac function and climbing ability; (3) determine whether Cg-Gal4-mediated *miR-276a* manipulation is associated with changes in *miR-276a* and *nej* expression in cardiac samples, cardiac function, and climbing ability; (4) evaluate the effects of exercise on cardiac function, climbing ability, and *miR-276a* and *nej* expression in cardiac samples following Hand-Gal4- and Cg-Gal4-mediated *miR-276a* manipulation.

## 2. Results

### 2.1. Altered Cardiac nej Expression Impairs Cardiac Function in Drosophila

Cardiac *nej* expression, cardiac function, and climbing ability were analyzed using one-way ANOVA. The complete omnibus statistics, including degrees of freedom, F values, exact *p* values, and effect sizes, are presented in Table 1. Multiplicity-adjusted pairwise *p* values and 95% confidence intervals are presented in Table 2.

**Figure 1:**
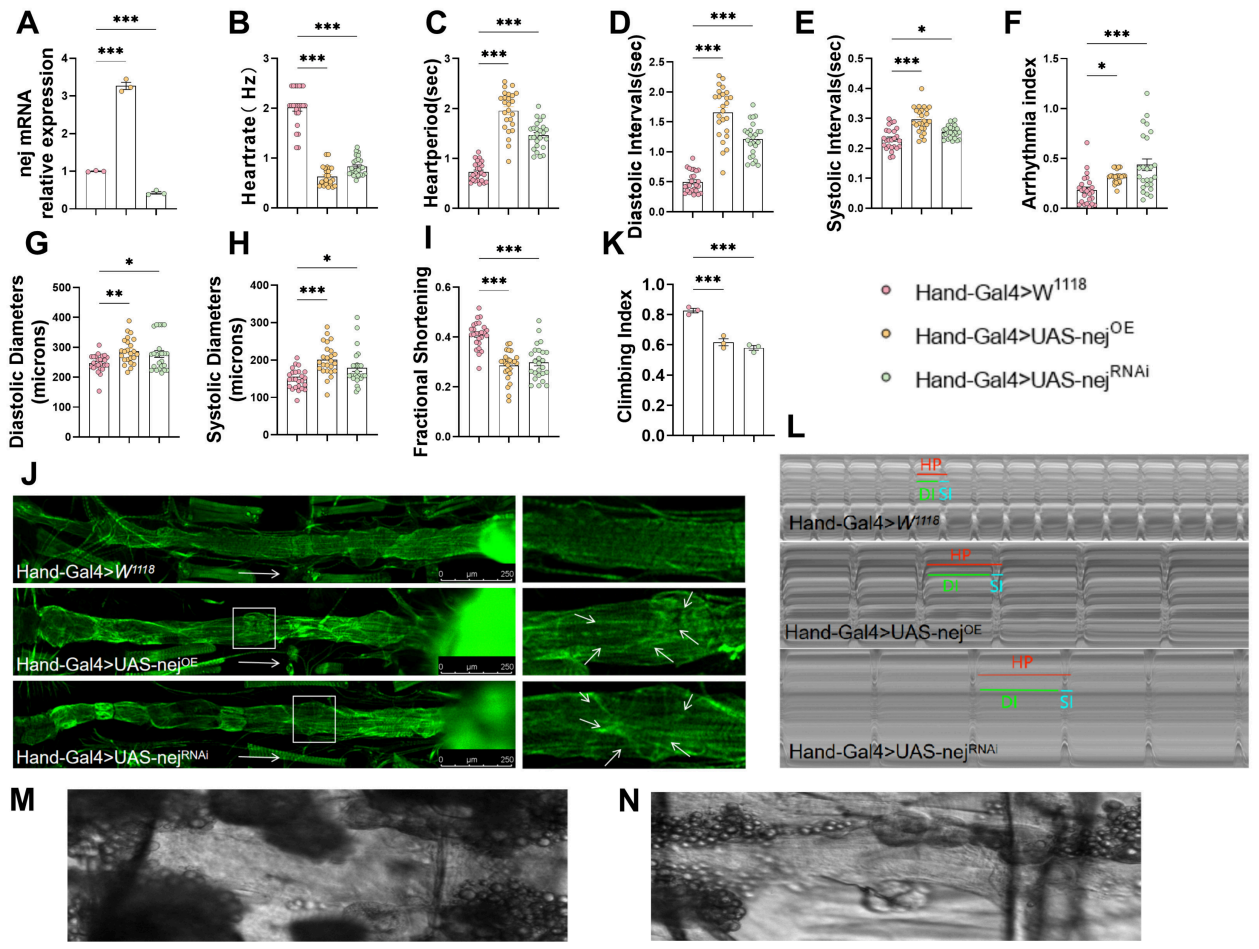
Hand-Gal4 > W^1118^ represents the Hand-Gal4 driver control, Hand-Gal4 > UAS-nej^OE^ is the group with heart-specific overexpression of *nej*, Hand-Gal4 > UAS-nej^RNAi^ is the group with heart-specific knockdown of *nej*. (**A**) Expression level of *nej* in the heart (*N* = 3 independent biological replicates; 25–35pooled hearts per replicate). (**B**–**I**) denotes heart rate, heart period, diastolic interval, systolic interval, arrhythmia index, diastolic diameter, systolic diameter, fractional shortening, respectively (*N* = 25–35 individual *Drosophila*). (**J**) Phalloidin staining image of *Drosophila* myocardial cytoskeleton. White boxes mark areas of disorganized and relaxed myofibrils. The small image on the side shows the area marked by the white box. White arrows indicate the direction towards the *Drosophila’s* head. Scale bar = 250 μm. (**K**) *Drosophila* climbing index (*N* = 3 independent biological replicates; each replicate consisted of one independently tested group of 60 flies). (**L**) M-mode electrocardiogram, with a recording time of 10 s per segment. Horizontal green line: diastolic interval, horizontal blue line: systolic interval, horizontal red line: heart period. (**M**) Heart with fat still attached. (**N**) A Heart with the fat removed * *p* < 0.05, ** *p* < 0.01, *** *p* < 0.001.

RT-qPCR showed that cardiac-specific overexpression or knockdown of *nej* significantly increased or decreased *nej* expression, respectively (Figure 1A), confirming effective cardiac-specific manipulation of *nej* expression. Cardiac function assays showed that both *nej* upregulation and downregulation reduced heart rate (Figure 1B), prolonged heart period, diastolic interval and systolic interval (Figure 1C–E,L), and increased the arrhythmia index (Figure 1F). For pumping function, both manipulations increased diastolic and systolic diameters (Figure 1G,H) and decreased fractional shortening (Figure 1I). Phalloidin staining showed that both *nej* upregulation and downregulation disrupted myocardial fiber organization and produced a loosened fiber arrangement (Figure 1J). Climbing assays further showed that both conditions reduced climbing ability in *Drosophila* (Figure 1K).

### 2.2. miR-276a Interacts with the nej 3′ Untranslated Region, and Cardiac miR-276a Manipulation Alters nej mRNA Expression and Cardiac Function

Normalized luciferase activity in 293T cells was analyzed using a two-way ANOVA, with reporter construct and mimic treatment included as fixed factors. The model included the main effects of reporter construct and mimic treatment, as well as the reporter construct × mimic treatment interaction (Table 3 and Table 4). Cardiac *miR-276a* and *nej* mRNA expression, cardiac function, as well as climbing ability were analyzed using one-way ANOVA. The complete omnibus statistics, including degrees of freedom, F values, exact *p*-values, and effect sizes, are presented in Table 5. Multiplicity-adjusted pairwise *p* values and 95% confidence intervals are presented in Table 6.

**Figure 2:**
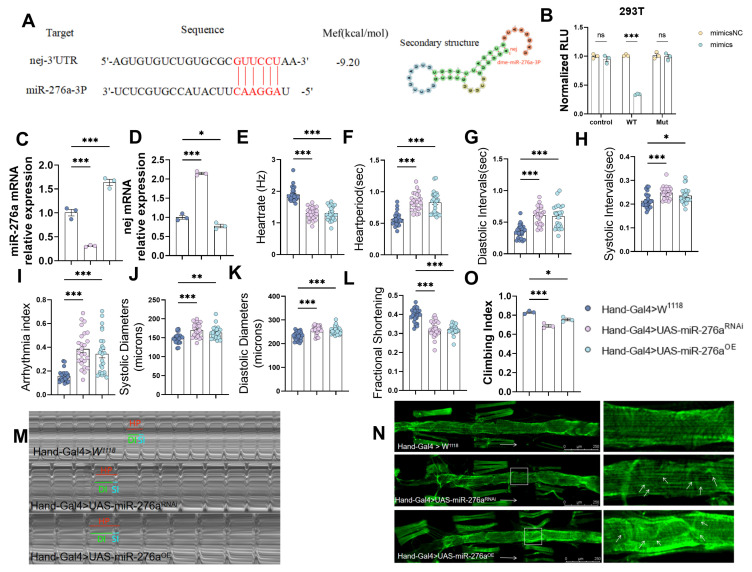
Hand-Gal4 > W^1118^ represents the Hand-Gal4 driver control group, Hand-Gal4 > UAS-miR-276a^RNAi^ is the group with heart-specific knockdown of *miR-276a*, Hand-Gal4 > UAS-miR-276a^OE^ is the group with heart-specific overexpression of *miR-276a*. (**A**) Prediction results from the TargetScanFly online software (v6.2). (**B**) Dual-luciferase reporter gene assay, three independent transfection experiments were performed (*N* = 3, and each data point represents one independent transfection). (**C**) Expression level of *miR-276a* in the heart (*N* = 3 independent biological replicates; each replicate consisted of a pooled sample of 25–35 hearts). (**D**) Expression level of *nej* in the heart (*N* = 3 independent biological replicates; each replicate consisted of a pooled sample of 25–35 hearts). (**E**–**L**) Heart rate, heart period, diastolic interval, systolic interval, arrhythmia index, systolic diameter, diastolic diameter, fractional shortening, respectively (*N* = 25–35 individual *Drosophila* per group; each data point represents one *Drosophila* as the experimental unit). (**M**) M-mode electrocardiogram, with a recording time of 10 s per segment. Horizontal green line: diastolic interval, horizontal blue line: systolic interval, horizontal red line: heart period. (**N**) Phalloidin staining image of *Drosophila* myocardial cytoskeleton. White boxes mark areas of disorganized and relaxed myofibrils. The small image on the side shows the area marked by the white box. White arrows indicate the direction towards the *Drosophila’s* head. Scale bar = 250 μm. (**O**) *Drosophila* climbing index (*N* = 3 independent biological replicates; each replicate consisted of one independently tested group of 60 flies). * *p* < 0.05, ** *p* < 0.01, *** *p* < 0.001.

TargetScanFly analysis identified a putative *miR-276a* binding site at positions 1567–1573 of the *nej* 3′ untranslated region (Figure 2A). In vitro dual-luciferase reporter assays showed that co-transfection with *miR-276a* mimics significantly reduced luciferase activity (Figure 2B), supporting a functional interaction between *miR-276a* and the *nej* 3′ untranslated region. We then used the UAS-Gal4 system to generate *Drosophila* with specific knockdown and overexpression of *miR-276a* in the heart. RT-qPCR analysis revealed that, compared with the control group, *miR-276a* expression levels were significantly reduced in the heart-specific knockdown group, whereas they were significantly elevated in the overexpression group (Figure 2C). In the knockdown group, the expression level of *nej* in the heart was significantly elevated, whereas in the overexpression group, it was significantly reduced (Figure 2C), suggesting that *miR-276a* negatively regulates *nej* expression. Cardiac function assays showed that both *miR-276a* knockdown and overexpression reduced heart rate (Figure 2D), prolonged heart period, diastolic interval and systolic interval (Figure 2F–H,M), and increased the arrhythmia index (Figure 2I). With regard to cardiac pumping function, both groups of *Drosophila* exhibited a significant increase in both diastolic diameter and systolic diameter (Figure 2K,J) and a decrease in fractional shortening (Figure 2L). Phalloidin staining results showed that both upregulation and downregulation of *miR-276a* led to myofibril disorganization and loose arrangement in the *Drosophila* heart (Figure 2N). It is worth noting that the cardiac structural and functional abnormalities caused by the altered expression of *miR-276a* are similar to the phenotype induced by altered expression of *nej*. Climbing ability tests revealed that both heart-specific knockdown and overexpression of *miR-276a* resulted in a significant reduction in the climbing ability of the *Drosophila* (Figure 2O).

### 2.3. Manipulation of miR-276a in Cg-Gal4-Expressing Tissues Alters Cardiac miR-276a/nej Expression and Cardiac Function

To investigate whether the changes in *miR-276a* expression in the fat body are associated with changes in cardiac *miR-276a*/*nej* expression and cardiac function, Cg-Gal4 *Drosophila* were crossed with UAS-miR-276a^RNAi^ or UAS-miR-276a^OE^ *Drosophila*. miR-276a expression in the fat body and heart, cardiac *nej* mRNA expression, cardiac function parameters, and climbing ability were analyzed using one-way ANOVA. Complete omnibus statistics, including degrees of freedom, F values, exact *p*-values, and effect sizes, are presented in Table 7. Multiplicity-adjusted pairwise *p* values and 95% confidence intervals are presented in Table 8.

**Figure 3:**
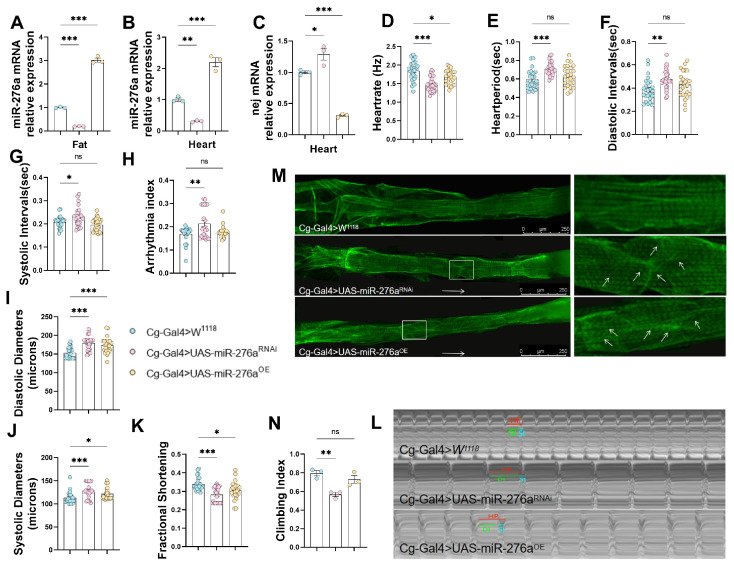
Cg-Gal4 > W^1118^ represents the Cg-Gal4 driver control group, Cg-Gal4 > UAS-miR-276a^RNAi^ represents the group with specific knockdown of *miR-276a* in adipose tissue, and Cg-Gal4 > UAS-miR-276a^OE^ represents the group with specific overexpression of *miR-276a* in adipose tissue. (**A**) Expression level of *miR-276a* in adipose tissue (*N* = 3, each point represents adipose tissue from 25 to 35 *Drosophila*, 3 independent biological replicates). (**B**) Expression level of *miR-276a* in the heart (*N* = 3 independent biological replicates; each replicate consisted of a pooled sample of 25–35 hearts). (**C**) Expression level of *nej* in the heart (*N* = 3 independent biological replicates; each replicate consisted of a pooled sample of 25–35 hearts). (**D**–**K**) Heart rate, heart period, diastolic interval, systolic interval, arrhythmia index, diastolic diameter, systolic diameter, fractional shortening, respectively (*N* = 25–35 individual *Drosophila* per group; each data point represents one *Drosophila* as the experimental unit). (**L**) M-mode electrocardiogram, with a recording time of 10 s per segment. Horizontal green line: diastolic interval, horizontal blue line: systolic interval, horizontal red line: heart period. (**M**) Phalloidin staining image of *Drosophila* myocardial cytoskeleton. White boxes mark areas of disorganized and relaxed myofibrils. The small image on the side shows the area marked by the white box. White arrows indicate the direction towards the *Drosophila’s* head. Scale bar = 250 μm. (**N**) *Drosophila* climbing index (*N* = 3 independent biological replicates; each replicate consisted of one independently tested group of 60 flies). * *p* < 0.05, ** *p* < 0.01, *** *p* < 0.001.

Cg-Gal4-driven knockdown of *miR-276a* significantly reduced *miR-276a* expression in both the fat body and heart, whereas Cg-Gal4-driven overexpression significantly increased *miR-276a* expression in both tissues compared with the driver control group (Figure 3A,B). Cardiac *nej* expression showed the opposite pattern, with a significant increase in the *miR-276a* knockdown group and a significant decrease in the *miR-276a* overexpression group (Figure 3C).

Cardiac function assays showed that both *miR-276a* knockdown and overexpression significantly reduced heart rate compared with the driver control group (Figure 3D). However, changes in cardiac cycle parameters were more pronounced following *miR-276a* knockdown. Heart period, diastolic interval, and systolic interval were significantly prolonged in the knockdown group, whereas these parameters did not differ significantly between the overexpression and driver control groups (Figure 3E–G). Similarly, the arrhythmia index significantly increased in the knockdown group but remained unchanged in the overexpression group relative to the driver control group (Figure 3H). Representative cardiac M-mode traces are shown in Figure 3L. Regarding cardiac pumping function, diastolic diameter was significantly increased in both the *miR-276a* knockdown and overexpression groups (Figure 3I). Systolic diameter was significantly increased in the knockdown group, whereas the increase in the overexpression group did not reach statistical significance (Figure 3J). Fractional shortening was significantly reduced in both manipulation groups compared with the driver control group (Figure 3K). Phalloidin staining showed that both Cg-Gal4-driven knockdown and overexpression of *miR-276a* were associated with a sparser and more disorganized arrangement of myocardial fibers (Figure 3M). Climbing ability was significantly reduced in the *miR-276a* knockdown group, whereas the reduction observed in the overexpression group was not statistically significant compared with the driver control group (Figure 3N).

Collectively, these findings indicate that altering *miR-276a* expression in Cg-Gal4-expressing tissues, including the fat body, is associated with changes in cardiac *miR-276a*/*nej* expression, cardiac structure and function, and climbing ability. These abnormalities were generally more pronounced following *miR-276a* knockdown than following overexpression.

### 2.4. Exercise Improves Cardiac Function in Drosophila with Altered Cardiac miR-276a Expression

**Figure 4:**
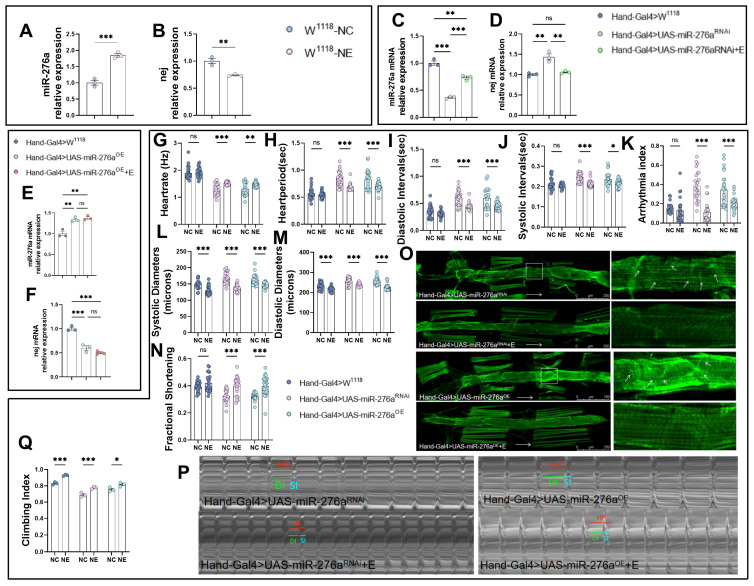
The W^1118^ group is the normal sedentary group, W^1118^ + E group is the exercise group. Hand-Gal4 > W^1118^ is the Hand-Gal4 driver control group, Hand-Gal4 > UAS-miR-276a^RNAi^ is the heart-specific *miR-276a* knockdown group, Hand-Gal4 > UAS-miR-276a^RNAi^ + E is the heart-specific *miR-276a* knockdown exercise group, Hand-Gal4 > UAS-miR-276a^OE^ is the heart-specific *miR-276a* overexpression group, and Hand-Gal4 > UAS-miR-276a^OE^ + E is the heart-specific *miR-276a* overexpression exercise group. (**A**) Expression level of wild-type *Drosophila miR-276a* in the heart (*N* = 3 independent biological replicates; each replicate consisted of a pooled sample of 25–35 hearts). (**B**) Expression level of *nej* in the heart of wild-type *Drosophila* (*N* = 3 independent biological replicates; each replicate consisted of a pooled sample of 25–35 hearts). (**C**) Knockdown group and knockdown exercise group expression levels of *miR-276a* in the heart (*N* = 3 independent biological replicates; each replicate consisted of a pooled sample of 25–35 hearts). (**D**) Knockdown and knockdown exercise group *nej* expression levels in the heart (*N* = 3 independent biological replicates; each replicate consisted of a pooled sample of 25–35 hearts). (**E**) Expression levels of *miR-276a* in the heart of the overexpression and overexpression exercise groups (*N* = 3 independent biological replicates; each replicate consisted of a pooled sample of 25–35 hearts). (**F**) Overexpression and overexpression exercise group *nej* expression levels in the heart (*N* = 3 independent biological replicates; each replicate consisted of a pooled sample of 25–35 hearts). (**G**–**N**) Heart rate, heart period, diastolic interval, systolic interval, arrhythmia index, diastolic diameter, systolic diameter, fractional shortening, respectively (*N* = 25–35 individual *Drosophila* per group; each data point represents one *Drosophila* as the experimental unit). (**O**) Phalloidin staining image of *Drosophila* myocardial cytoskeleton. White boxes mark areas of disorganized and relaxed myofibrils. The small image on the side shows the area marked by the white box. White arrows indicate the direction towards the *Drosophila ’s* head. Scale bar = 250 μm. (**P**) M-mode electrocardiogram, with a recording time of 10 s per segment. Horizontal green line: diastolic interval, horizontal blue line: systolic interval, horizontal red line: heart period. (**Q**) *Drosophila* climbing index (*N* = 3 independent biological replicates; each replicate consisted of one independently tested group of 60 flies). * *p* < 0.05, ** *p* < 0.01, *** *p* < 0.001.

To investigate the regulatory role of exercise on the *miR-276a/nej* expression in the heart, we first subjected wild-type *Drosophila* to a one-week exercise intervention. RT-qPCR analysis showed that exercise significantly increased cardiac *miR-276a* expression and decreased *nej* expression (Figure 4A,B; Table 9). The same exercise intervention was subsequently applied to *Drosophila* with cardiac-specific knockdown or overexpression of *miR-276a*, together with the corresponding driver control. Gene expression, cardiac function, and climbing ability were analyzed using two-way ANOVA, with genotype and exercise intervention included as fixed factors. Significant main effects of genotype and exercise were detected for *miR-276a* and *nej* mRNA expression and for all cardiac function parameters and climbing ability examined. Significant genotype × exercise interactions were observed for *miR-276a* and *nej* expression, heart rate, heart period, diastolic interval, systolic interval, arrhythmia index, systolic diameter, and fractional shortening. In contrast, the interactions were not significant for diastolic diameter or climbing ability. Complete model statistics, including degrees of freedom, F values, *p*-values, and partial η^2^, are presented in Table 10.

Follow-up simple-effects analyses showed exercise intervention led to an increase in heart *miR-276a* expression levels (Figure 4C) and a decrease in *nej* expression levels (Figure 4D). In the group with heart *miR-276a* overexpression, however, there were no significant changes in the expression levels of *miR-276a* and *nej* following exercise intervention (Figure 4E,F).

Cardiac function assays revealed that, with regard to cardiac rhythm, both the *miR-276a* knockdown and overexpression groups exhibited increased heart rate (Figure 4G), significantly shortened heart period, diastolic interval, and systolic interval (Figure 4H–J,P), and a significant reduction in the arrhythmia index (Figure 4K). With regard to cardiac pumping function, the systolic and diastolic diameters were significantly reduced in the heart *miR-276a* knockdown and overexpression groups (Figure 4L,M), the fractional shortening of the heart was significantly improved (Figure 4N). Phalloidin staining revealed that the disorganized arrangement and loose structure of the cardiac muscle fibers, which had originally been caused by upregulation or downregulation of *miR-276a*, were improved, with the cardiac muscle fibers becoming more tightly and neatly arranged (Figure 4O). The climbing ability test revealed that the climbing ability of *Drosophila* in all experimental groups was significantly improved compared with those in the control groups (Figure 4Q). Multiplicity-adjusted *p* values and 95% confidence intervals for all simple-effects comparisons are presented in Table 10. These results suggest that exercise improves cardiac and climbing defects associated with altered cardiac *miR-276a* expression, with clear modulation of the *miR-276a*/*nej* pathway observed in the knockdown condition.

### 2.5. Exercise Ameliorates Cardiac Dysfunction Induced by Cg-Gal4-Driven miR-276a Dysregulation in Drosophila

To examine exercise-related cardiac responses following Cg-Gal4-mediated *miR-276a* manipulation, *Drosophila* with Cg-Gal4-driven knockdown or overexpression of *miR-276a* were subjected to a one-week exercise intervention. M*iR-276a* expression in the fat body and heart and cardiac *nej* mRNA expression, cardiac function parameters, and climbing ability were analyzed using two-way ANOVA, with genotype and exercise intervention included as fixed factors. Significant genotype × exercise interactions were detected for *miR-276a* expression in the fat body and heart, heart rate, arrhythmia index, diastolic diameter, and climbing ability, whereas the interactions were not significant for *nej* expression, heart period, diastolic interval, systolic interval, systolic diameter, or fractional shortening. Complete model statistics, including degrees of freedom, F values, exact *p*-values, and partial η^2^, are presented in Table 11. Multiplicity-adjusted simple-effects comparisons and 95% confidence intervals are presented in Table 12.

**Figure 5:**
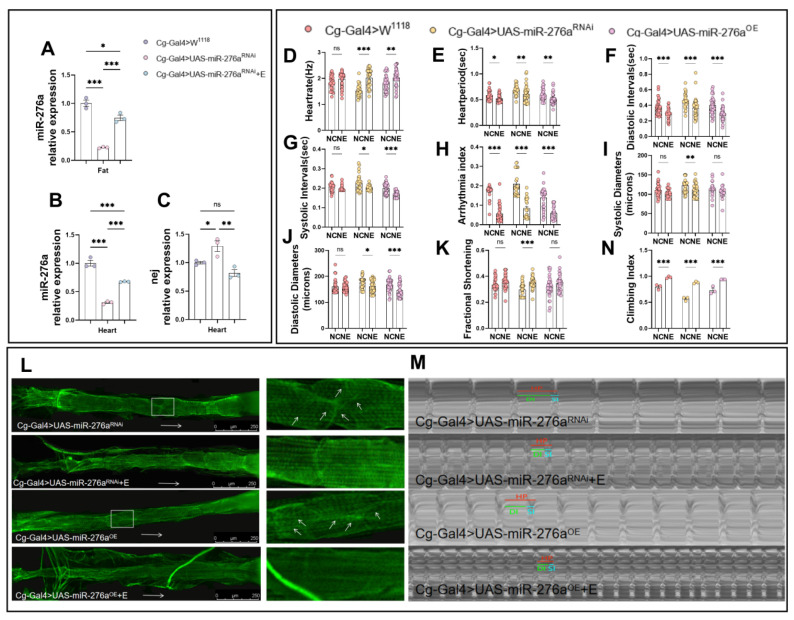
Cg-Gal4 > W^1118^ represents the Cg-Gal4 driver control group, Cg-Gal4 > UAS-miR-276a^RNAi^ represents the group with specific knockdown of *miR-276a* in adipose tissue, Cg-Gal4 > UAS-miR-276a^RNA*i*^ + E represents the group with specific knockdown of *miR-276a* in adipose tissue and exercise, Cg-Gal4 > UAS-miR-276a^OE^ represents the group with specific overexpression of *miR-276a* in adipose tissue, Cg-Gal4 > UAS-miR-276a^OE^ + E represents the group with specific overexpression of *miR-276a* in adipose tissue and exercise. (**A**) Expression levels of *miR-276a* in adipose tissue before and after exercise (*N* = 3, each point represents adipose tissue from 25 to 35 *Drosophila*, 3 independent biological replicates). (**B**) Expression levels of *miR-276a* in the heart before and after exercise (*N* = 3 independent biological replicates; each replicate consisted of a pooled sample of 25–35 hearts). (**C**) Expression levels of *nej* in the heart before and after exercise (*N* = 3 independent biological replicates; each replicate consisted of a pooled sample of 25–35 hearts). (**D**–**K**) Heart rate, heart period, diastolic interval, systolic interval, arrhythmia index, systolic diameter, diastolic diameter, fractional shortening, respectively (*N* = 25–35 individual *Drosophila* per group; each data point represents one *Drosophila* as the experimental unit). (**L**) Phalloidin staining image of *Drosophila* myocardial cytoskeleton after exercise. White boxes indicate abnormal regions. The small image on the side shows the area marked by the white box. white arrows point towards the head of the *Drosophila*. Scale bar = 250 μm. (**M**) M-mode electrocardiogram, with each segment representing 10 s. Horizontal green line: diastolic interval, horizontal blue line: systolic interval, horizontal red line: heart period. (**N**) Exercise capacity of each group before and after exercise (*N* = 3 independent biological replicates; each replicate consisted of one independently tested group of 60 flies). * *p* < 0.05, ** *p* < 0.01, *** *p* < 0.001.

Follow-up simple-effects analyses showed that exercise significantly increased *miR-276a* expression in both the fat body and heart in the driver control and *miR-276a* knockdown groups (Figure 5A,B). Exercise also significantly reduced cardiac *nej* expression in both genotypes (Figure 5C).

Cardiac function analyses showed that exercise significantly increased heart rate in the driver control, *miR-276a* knockdown, and *miR-276a* overexpression groups, although the significant genotype × exercise interaction indicated that the magnitude of this response differed among genotypes (Figure 5D). Exercise also shortened heart period and diastolic interval in all three genotypes (Figure 5E,G). For systolic interval, the exercise-induced reduction reached statistical significance in the *miR-276a* knockdown and overexpression groups but not in the driver control group; however, the genotype × exercise interaction was not significant (Figure 5F). Exercise significantly reduced the arrhythmia index in all three genotypes, with the magnitude of the reduction varying among genotypes (Figure 5H). Representative cardiac M-mode traces are shown in Figure 5M.

Regarding cardiac pumping function, exercise significantly reduced diastolic diameter in the *miR-276a* knockdown and overexpression groups but not in the driver control group, consistent with the significant genotype × exercise interaction (Figure 5J). Exercise exerted significant overall effects on systolic diameter and fractional shortening, although their genotype × exercise interactions were not significant. Adjusted within-genotype comparisons showed that systolic diameter was significantly reduced and fractional shortening was significantly increased in the *miR-276a* knockdown group, whereas the corresponding changes did not reach statistical significance in the driver control or *miR-276a* overexpression groups (Figure 5I,K). These differences in within-genotype significance should not, however, be interpreted as evidence of different exercise responses because the interaction terms were not significant.

Phalloidin staining qualitatively showed that exercise alleviated the sparse and disorganized myocardial fiber arrangement associated with Cg-Gal4-driven *miR-276a* knockdown or overexpression (Figure 5L). Climbing ability showed a significant genotype-dependent response to exercise. Exercise increased climbing ability in the driver control and *miR-276a* knockdown groups but decreased climbing ability in the *miR-276a* overexpression group (Figure 5N).

Collectively, these findings indicate that exercise modifies cardiac *miR-276a*/*nej* expression and cardiac function in Cg-Gal4/UAS *miR-276a* transgenic *Drosophila*. Improvements in cardiac pumping function were more consistently detected under *miR-276a* knockdown conditions, whereas the opposite climbing ability in the overexpression group indicates that the effects of exercise depend on *miR-276a* expression status.

## 3. Discussion

This study examined the relationships among *miR-276a*, *nej*, cardiac function, Cg-Gal4-mediated *miR-276a* manipulation, and exercise-related functional changes in *Drosophila*. Both cardiac-specific overexpression and knockdown of *nej* impaired cardiac rhythm and pumping function and disrupted myocardial fiber organization. Cardiac-specific manipulation of *miR-276a* produced similar phenotypes, while the dual-luciferase reporter assay supported an interaction between *miR-276a* and the *nej* 3′ untranslated region, and RT-qPCR results were consistent with negative regulation of *nej* mRNA by *miR-276a*. Exercise improved cardiac and climbing ability in both cardiac-specific *miR-276a* knockdown and overexpression groups. In the cardiac knockdown group, exercise increased *miR-276a* expression and decreased *nej* expression in cardiac samples, whereas neither transcript changed significantly in the cardiac overexpression group despite functional improvement. Cg-Gal4-mediated *miR-276a* manipulation was associated with altered *miR-276a* and *nej* expression in cardiac samples and with cardiac structural and functional phenotypes. Exercise also improved cardiac function following Cg-Gal4-mediated *miR-276a* knockdown and overexpression. Under Cg-Gal4-mediated knockdown conditions, these improvements were accompanied by increased *miR-276a* and decreased *nej* expression in cardiac samples. Collectively, these findings indicate that exercise modulates cardiac function and *miR-276a*/*nej* expression in a context-dependent manner and suggest a possible contribution of the fat body or other Cg-Gal4-expressing tissues.

CBP/p300 is a core member of the histone acetyltransferase family [23] and plays an important role in maintaining histone acetylation homeostasis [24,25,26]. In *Drosophila*, its direct homolog is *nej* [27]. Previous studies have shown that increased CBP/p300 expression induces myocardial hypertrophy in mice, whereas inhibiting its expression/activity alleviates myocardial hypertrophy [9,28], suggesting that abnormal *CBP/p300* expression may be associated with cardiac dysfunction. In the present study, cardiac-specific overexpression of *nej* in *Drosophila* caused rhythm dysfunction, including reduced heart rate and increased arrhythmia index, as well as pumping dysfunction, including enlarged diastolic and systolic diameters and reduced fractional shortening. Increased diastolic diameter may reflect dilation-like cardiac remodeling, whereas increased systolic diameter indicates impaired contractile function [29]. Notably, cardiac-specific knockdown of *nej* produced similar cardiac dysfunction and disrupted myocardial fiber organization. The myocardial fiber disorganization caused by altered *nej* expression is consistent with previous studies linking CBP/p300-related regulation to myocardial structure and function [30,31,32,33]. The observation that both increased and decreased *nej* expression produced cardiac abnormalities is compatible with the possibility that cardiac function is sensitive to deviations in *nej* expression.

As a key transcriptional co-activator, *CBP/p300* is involved in a variety of biological processes, including the cell cycle, proliferation, differentiation, apoptosis, and DNA damage repair [34,35,36,37,38]. We, therefore, speculate that reduced *nej* expression may impair cardiac structure and function by weakening transcriptional regulation of these fundamental biological processes. Conversely, increased *nej* expression may lead to excessive acetylation-related activity or downstream transcriptional activation, thereby promoting pathological cardiac remodeling. These mechanisms remain to be tested by measuring acetylation levels and downstream transcriptional targets. Together, our findings indicate that both increased and decreased cardiac *nej* expression impair cardiac rhythm and pumping function.

The bidirectional cardiac defects caused by *nej* overexpression and knockdown are compatible with the possibility that cardiac function is sensitive to deviations in *nej* expression. Post-transcriptional regulation, particularly miRNA-mediated control of target *mRNAs*, may contribute to gene-expression balance and biological robustness [39,40]. We therefore investigated miRNA-mediated regulation upstream of *nej*. Previous genetic and reporter analyses identified *nej* as a downstream target of the FMRP-*miR-276a* regulatory pathway in *Drosophila* C4da neurons [13]. In this study, bioinformatic prediction and the in vitro dual-luciferase reporter assay supported an interaction between *miR-276a* and the *nej* 3′ untranslated region. Cardiac *miR-276a* knockdown was accompanied by increased *nej* mRNA expression, whereas cardiac *miR-276a* overexpression was accompanied by decreased *nej* mRNA expression. These results are consistent with negative regulation of *nej* mRNA by *miR-276a* in cardiac samples. In vivo cardiac function assays revealed that both upregulation and downregulation of cardiac *miR-276a* expression led to cardiac arrhythmias, cardiac pumping dysfunction, and reduced climbing ability. These phenotypes resembled those caused by altered *nej* expression. However, *miR-276a* may regulate multiple genes involved in myocardial structure and function, while altered *nej* expression may affect numerous downstream transcriptional processes. The observed phenotypes may therefore reflect a broader regulatory network rather than the effect of a single molecular relationship.

Beyond local cardiac regulation, adipose tissue may provide an additional source of miRNAs that influence this pathway. Adipose tissue is an important source of circulating miRNAs and can regulate gene expression in distant organs through miRNA-mediated inter-organ communication [41]. Previous studies have reported that *miR-276a* is enriched in adipose tissue [42]. We, therefore, examined whether Cg-Gal4-mediated *miR-276a* manipulation was associated with changes in *miR-276a* and *nej* expression in cardiac samples and with cardiac function. In the present study, Cg-Gal4-mediated *miR-276a* knockdown and overexpression were associated with cardiac rhythm abnormalities, pumping dysfunction, myocardial fiber disorganization, and impaired climbing ability. These phenotypes resembled those observed following cardiac-specific manipulation of *miR-276a* and *nej*. RT-qPCR further showed that Cg-Gal4-mediated *miR-276a* knockdown and overexpression were associated with corresponding changes in *miR-276a* expression and opposite changes in *nej* expression in dissected cardiac samples. The observed associations, therefore, suggest a possible contribution of the fat body to cardiac regulation.

Heart-associated hemocytes and pericardial cells are increasingly recognized as potential contributors to insect cardiac physiology and immune responses. In mosquitoes, bacterial infection recruits periostial hemocytes to regions surrounding the heart, where hemocyte-derived nitric oxide contributes to infection-associated changes in heart rate [43,44,45]. Pericardial cells can also upregulate immune-related factors, including lysozyme, in response to immune stimulation [45]. These findings indicate that cardiac-associated cells can influence the cardiac microenvironment through immune and paracrine mechanisms. However, these observations were primarily obtained under immune-challenge conditions, and their relevance to cardiac regulation in *Drosophila* under the present experimental conditions remains unknown. Direct evidence for *miR-276a*/*nej* regulation in hemocytes or pericardial cells is currently lacking. Moreover, the dissected cardiac samples used in the present study may contain cardiomyocytes, pericardial cells, and heart-associated hemocytes. Therefore, the measured changes in *miR-276a* and *nej* expression cannot be attributed exclusively to cardiomyocytes. Future cell-type-specific expression analyses and genetic manipulations will be required to determine whether *miR-276a* and *nej* are regulated in these cardiac-associated cell populations and whether these cells contribute to the observed cardiac phenotypes.

Extracellular miRNA secretion and transport provide a possible mechanism for inter-tissue communication in *Drosophila*. *miR-276a*−3p has been detected in extracellular-vesicle and extracellular Ago−1-associated fractions from the conditioned media of *Drosophila* cells [46]. In addition, glia-derived exosomal miR−274 has been shown to enter the circulating hemolymph and regulate recipient neuronal and tracheal cells in vivo [47]. These observations support the biological plausibility of extracellular miRNA secretion, transport, and recipient-cell uptake in *Drosophila*. However, whether *miR-276a* is secreted from the fat body, circulates in the hemolymph, and is taken up by cardiac or cardiac-associated cells remains unknown. This study did not measure *miR-276a* in hemolymph or extracellular vesicle fractions or perturb miRNA secretion and uptake pathways. Therefore, extracellular transfer of *miR-276a* represents a hypothesis for future investigation rather than a mechanism established by the present data.

Exercise is an effective non-pharmacological intervention that improves cardiac function, supports adaptive cardiac remodeling and enhances exercise capacity [48,49,50]. Exercise has also been reported to alter miRNA expression in multiple tissues, suggesting that miRNA-mediated regulation may participate in exercise-induced cardiac adaptation [51,52,53,54]. In this study, a one-week exercise intervention increased cardiac *miR-276a* expression and decreased cardiac *nej* expression in wild-type *Drosophila*. In *Drosophila* with cardiac-specific *miR-276a* knockdown, exercise increased cardiac *miR-276a* expression, decreased *nej* expression, and improved cardiac phenotypes. However, in *Drosophila* with cardiac-specific *miR-276a* overexpression, exercise improved cardiac function without significantly changing *miR-276a* or *nej* expression. Changes in *miR-276a* and *nej* expression accompanied exercise-related cardiac improvement under cardiac *miR-276a* knockdown conditions but were not observed in the cardiac overexpression group despite functional improvement. This dissociation suggests that additional exercise-responsive mechanisms may contribute. Future studies should investigate other exercise-responsive pathways that may cooperate with or act independently of *miR-276a*/*nej* regulation.

Exercise also produced cardiac improvements following Cg-Gal4-mediated *miR-276a* manipulation. In Cg-Gal4-mediated *miR-276a* knockdown group, exercise significantly increased *miR-276a* expression in fat body and cardiac samples, decreased *nej* expression in cardiac samples, and improved cardiac function and climbing ability. In Cg-Gal4-mediated *miR-276a* overexpression group, exercise significantly improved most measured cardiac functional parameters, whereas fractional shortening did not change significantly. These context-dependent responses suggest that the fat body may contribute to the cardiac response to exercise.

The present findings also identify several priorities for future research. First, although the reporter assay and RT-qPCR results support a regulatory relationship between *miR-276a* and *nej*, future studies should combine cardiac genetic epistasis and rescue experiments with Nej protein quantification to determine whether *nej* mediates the cardiac phenotypes caused by *miR-276a* manipulation. Second, the bidirectional phenotypes are compatible with an optimal-range interpretation but require further validation. Graded manipulation of Gal4 activity, variation in transgene dosage, and adult-restricted temporal induction should be used to determine whether cardiac dysfunction scales with deviations of *miR-276a* and *nej* from their endogenous expression levels. Future experiments should also include corresponding UAS-only controls and appropriately matched genetic backgrounds to exclude insertion-site and stock-specific effects more completely. Third, the possible involvement of the fat body requires direct tissue-specific evidence. Future studies should characterize the adult expression domain of Cg-Gal4, verify cardiac sample enrichment and depletion of fat body markers, measure *miR-276a* in hemolymph and extracellular-vesicle fractions, and perturb miRNA secretion and uptake pathways to determine whether *miR-276a* is transferred from the fat body to cardiac or cardiac-associated cells. Finally, genetic decoupling experiments using a cardiac *miR-276a*-insensitive *nej* transgene should be combined with exercise intervention to determine whether *miR-276a*-dependent regulation of *nej* is necessary for exercise-related cardiac improvement and to identify additional exercise-responsive mechanisms.

## 4. Materials and Methods

### 4.1. Drosophila Strains and Grouping

**Figure 6:**
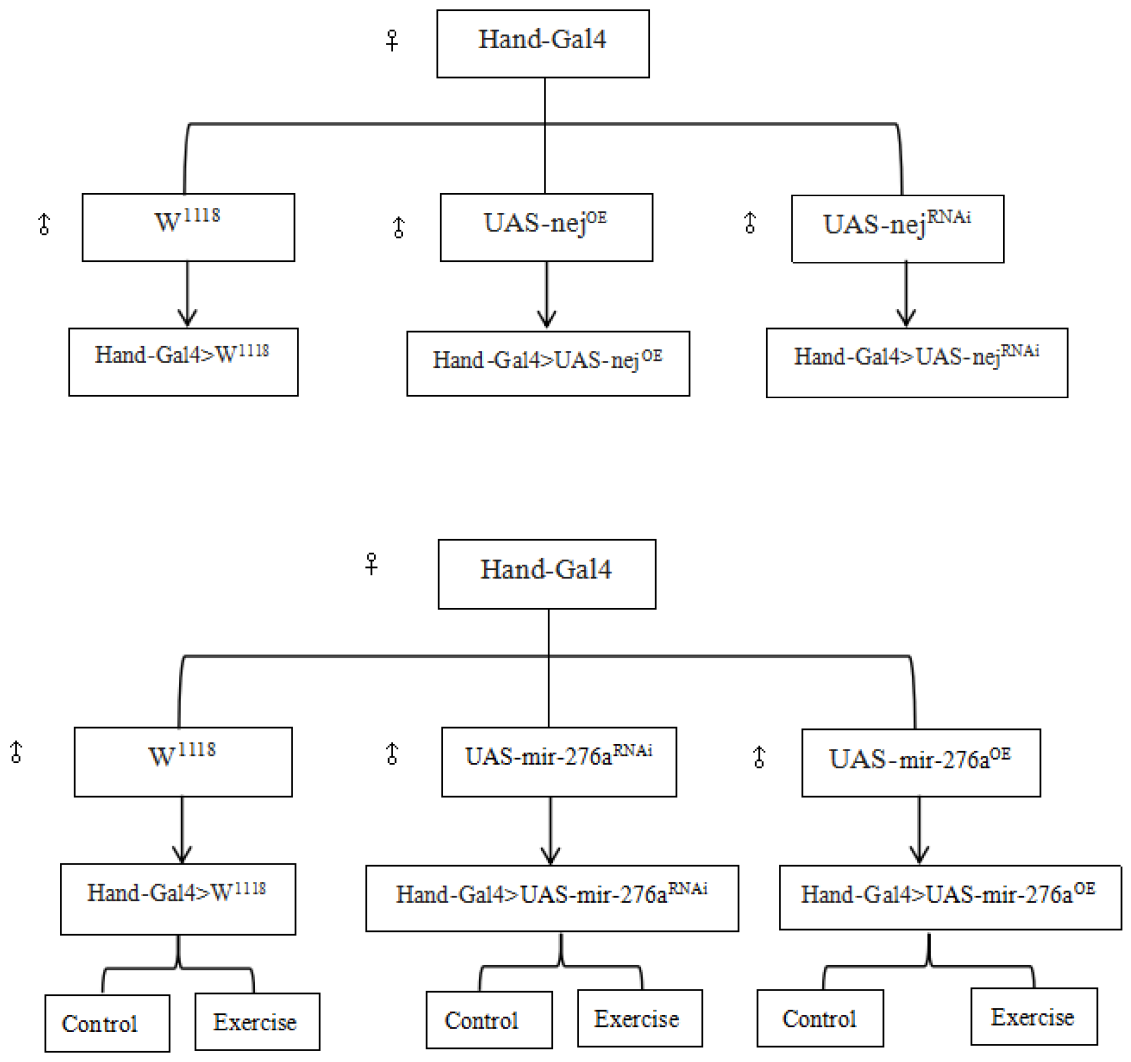

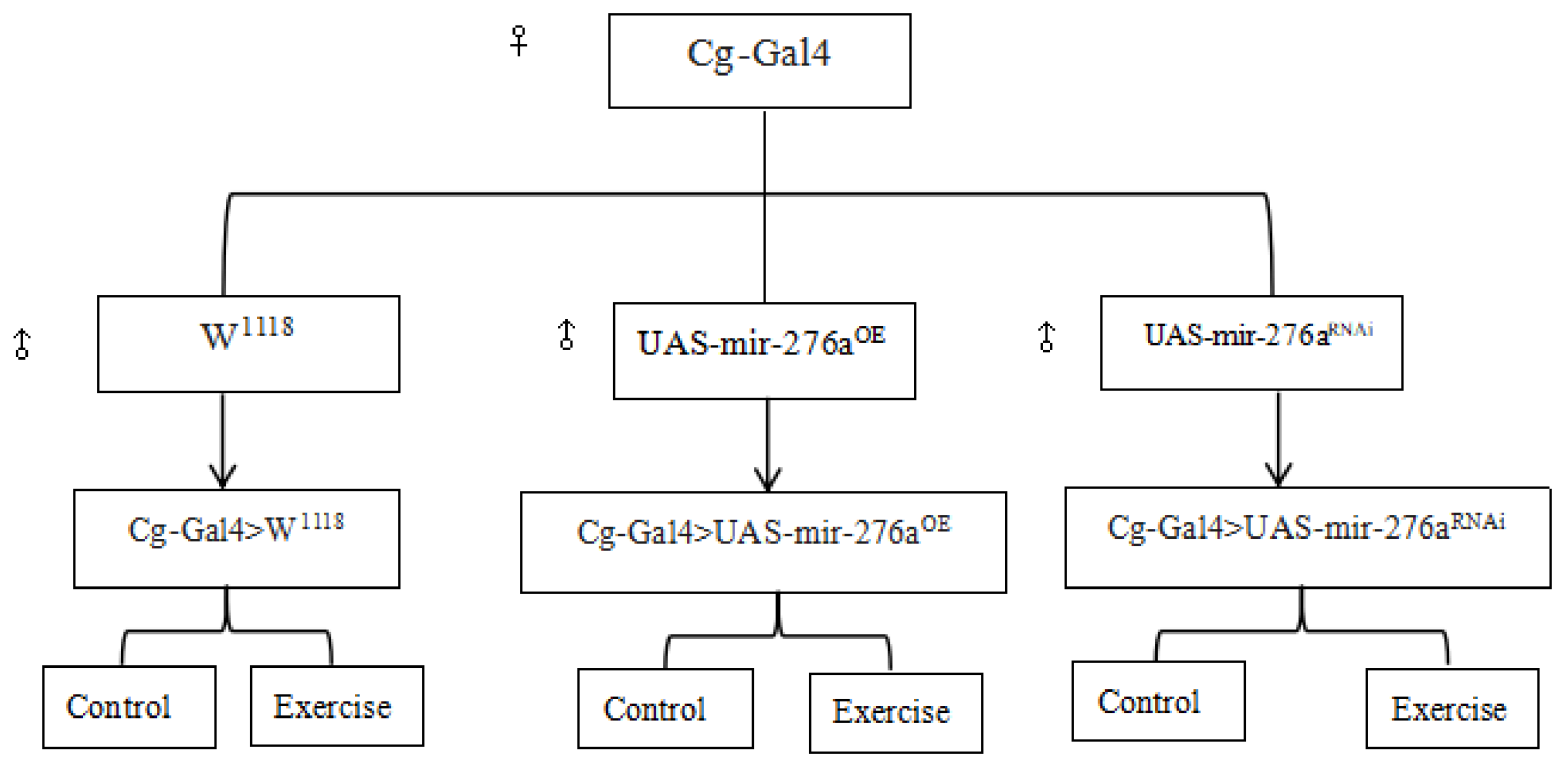
Schematic diagram of *Drosophila* hybridization.

The *Drosophila* strains used in this study are listed in Table 13, and the corresponding crossing strategy is illustrated in Figure 6. Virgin females were collected within 12 h of eclosion and crossed with the appropriate males. Virgin F1 offspring were subsequently collected within 12 h of eclosion and maintained until 10 days of age. All experiments were performed using 10-day-old virgin F1 *Drosophila*, which were assigned to the experimental groups listed in Table 14.

### 4.2. Exercise Devices and Exercise Schemes

**Figure 7:**
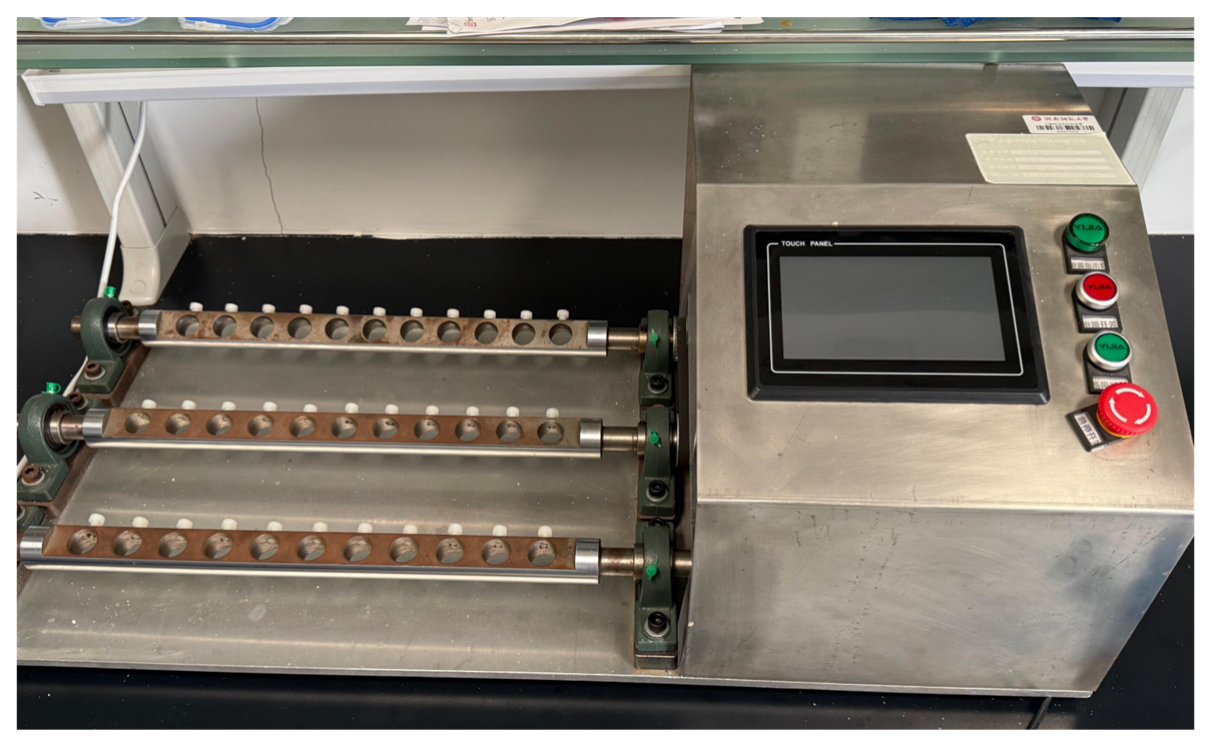
*Drosophila* exercise equipment.

Using exercise equipment in our laboratory, as shown in Figure 7, we induced *Drosophila* to move continuously upward by utilizing their characteristic anti-gravitational climbing behavior [55]. The environment during exercise is consistent with the external environment. Based on various exercise protocols and with appropriate modifications [56,57], the *Drosophila* were exercised using the apparatus shown in Figure 7. The apparatus was operated at a rotational speed of 0.91 rev/s, producing a vertical displacement amplitude of 10 cm. Thirty *Drosophila* were housed in each exercise tube. The experiments were conducted at 25 °C and 60–70% relative humidity under a 12 h light/12 h dark cycle, as listed in Table 15. The *Drosophila* in the exercise group began exercising on the second day after eclosion, exercising for 3 days, then resting for 1 day, and then exercising for another 3 days. The exercise program ended on the 9 day. The exercise was performed at the same time each day, lasting for 2.5 h. Various parameters were tested in each group of *Drosophila* the day after the exercise ended. Sedentary *Drosophila* were maintained in identical tubes under the same environmental conditions and were handled at the same times as the exercised *Drosophila* but were not subjected to repeated displacement.

### 4.3. Indicator Detection

#### 4.3.1. RT-qPCR Detection

##### *Drosophila* Heart Extraction

The *Drosophila* physiological solution was prepared in advance and a needle-drawing device was used to draw out a capillary blood-collection needle to the appropriate size; this is a necessary step for aspirating fat from the *Drosophila* body.

Procedure for Preparing a Semi-Exposed *Drosophila* Heart: First, artificial hemolymph was prepared and deoxygenated for 30 min at 26 °C. Next, the *Drosophila* was anesthetized with carbon dioxide for 2–3 min and secured to a Petri dish coated with medical petroleum jelly. Then, under a microscope, specialized scissors and forceps were used to remove the head, and oxygenated artificial hemolymph was added. Finally, under a microscope, specialized scissors and forceps were used to incise the abdominal cavity and the abdominal corona, and the internal organs were removed with forceps. A capillary needle was used to aspirate the surrounding fat, exposing the heart, which was then retrieved with forceps. It was placed into an EP tube containing PBS solution; each sample consists of 30 *Drosophila* hearts.

##### *Drosophila* Fat Tissue Extraction

First, the artificial hemolymph was prepared [58] and deoxygenated for 30 min at 26 °C. Next, the *Drosophila* was anesthetized with carbon dioxide for 2–3 min and secured to a Petri dish coated with medical-grade petroleum jelly. Then, under a microscope, specialized scissors and forceps were used to remove the head and the *Drosophila* was placed in oxygenated artificial hemolymph. Finally, under a microscope, special scissors and forceps were used to incise the abdominal cavity and the abdominal corona, and the internal organs were removed. Subsequently, forceps were used to extract the fat from the *Drosophila*’s abdomen before it was placed into an EP tube containing PBS solution, with fat from 30 *Drosophila* constituting one sample.

As described in the previous section, *Drosophila* heart and fat tissues were isolated and then centrifuged (4 °C, 7500 r/s, 12 min). After centrifugation, the precipitate was retained. Firstly, total RNA was extracted using Trizol (Trizol Invitrogen, Waltham, MA, USA) via organic solvent extraction. Two 3 mm grinding beads were added, then centrifuged three times for 40 s each time. The extracted sample was then placed into an inner tube and centrifuged three times. Then, 500 μL of wash solution was added, centrifuged for 60 s, before the wash solution was aspirated and discarded. This process was repeated three times. The elution buffer was added, performing three elution steps, before proceeding with reverse transcription. Reverse transcription was performed using PrimeScript (Takara Bio Inc., Kusatsu, Shiga, Japan) and miR-X (TaKaRa) kits. Three replicates of qPCR amplification reactions were conducted, with thermal cycling and fluorescence monitoring carried out on an ABI 7300 real-time PCR instrument (Applied Biosystems, Foster City, CA, USA). Real-time PCR was performed using SYBR Green (TaKaRa) standardized with *rp49/U6*. The *rp49* and *U6* genes were used as internal references to normalize the total RNA quantity. The gene primers are shown in Table 16:

#### 4.3.2. Preparation of Semi-Intact *Drosophila* Hearts and M-Mode Analysis

Following the previously described research method [58], prior to performing a semi-exposed surgery on *Drosophila*, the *Drosophila* physiological solution was prepared in advance and use a needle-drawing device to draw a capillary blood-collection needle to the appropriate size; this is a necessary step for aspirating fat from the *Drosophila* body.

Steps for preparing a semi-exposed *Drosophila* heart: First, an artificial hemolymph solution was prepared and deoxygenated for 30 min at 26 °C. Next, the *Drosophila* was anesthetized with carbon dioxide for 2–3 min and secured to a Petri dish coated with medical-grade petroleum jelly. Then, under a microscope, specialized scissors and forceps were used to remove the head, and oxygenated artificial hemolymph was added. Finally, under a microscope, specialized scissors and forceps were used to incise the abdominal cavity and the abdominal corona, and the internal organs were removed with forceps. A capillary needle was used to aspirate the surrounding fat, exposing the heart, and continuously pump oxygen into it for 30 min.

Note: Direct contact with the heart tube and the tail region of the heart tube was avoided, as the heart tube in the tail region is extremely unstable when fixed; this area should be completely avoided. Once the semi-exposed surgery on the *Drosophila* was complete, the solution was replaced with fresh physiological saline and prepared for optical scanning.

Then, a high-speed EM-CCD camera was used to record the heartbeat of *Drosophila* (video at 124 frames/second, 30 s) [59], and electrocardiogram data were recorded using HC Image Live software (v5.0.0). Semi-automatic optical heartbeat analysis is used to quantify heart rate, heart period, diastolic interval, systolic interval, and arrhythmia index, as well as diastolic diameter, systolic diameter, and fractional shortening.

#### 4.3.3. 293T Cell Culture and Dual-Luciferase Reporter Gene Assay

Human embryonic kidney 293T cells were cultured in complete growth medium consisting of high-glucose DMEM basal medium supplemented with 10% fetal bovine serum (FBS) and 1% penicillin-streptomycin (P/S) mixture, in a dedicated cell culture incubator set to a constant temperature of 37 °C, at a constant temperature of 37 °C, 24 h prior to the transfection experiment, 293T cells in the logarithmic growth phase were trypsinized and seeded at an appropriate density into 24-well cell culture plates to achieve a confluence of 70–80% at the time of transfection. Subsequently, using a liposomal transfection reagent (such as Lipofectamine 3000 or FuGENE HD), chemically synthesized small RNA oligonucleotide fragments—namely, *miR-276a* mimics (which mimic endogenous mature miRNAs in vivo) and the corresponding negative control mimics (NC mimics)—were co-transfected into 293T cells along with a dual-luciferase reporter vector carrying the target gene regulatory elements. The reporter vector, psiCheck−2 (synthesized and constructed by Tsingke Biotechnology, Beijing, China) has successfully cloned a fragment of the 3′ untranslated region (3′UTR) of the target gene *nej* into the multi-cloning site downstream of the fire *Drosophila* luciferase reporter gene; this plasmid also contains the sea-kidney luciferase gene as an internal control to correct for differences in transfection efficiency and cell counts between wells. After incubating the transfection complex at room temperature for 15–20 min, it was directly added to the cell culture medium in each well. The cell plates were then returned to the incubator for continued culture for 48 h, allowing the exogenous miRNA mimetic sufficient time to act on the 3′UTR region of the target mRNA and inhibit the downstream translation and expression of fire *Drosophila* luciferase. Upon completion of the transfection treatment, the culture supernatant was carefully aspirated from each well and the cells were gently washed twice with pre-chilled phosphate-buffered saline (PBS) to remove residual culture medium and serum-derived interfering components. An adequate volume of Passive Lysis Buffer was added to each well, and incubated on a room temperature shaker at low speed for 15–20 min to fully release the intracellularly expressed fire *Drosophila* luciferase and sea-kidney luciferase proteins. Finally, Promega’s Dual-Luciferase Reporter Assay System was used to sequentially inject fire *Drosophila* luciferase-specific substrate (luciferin, luciferin) sequentially on a luminometer equipped with an autosampler (or a multifunctional microplate reader with a luminescence detection module) to record the relative light units (RLU). Subsequently, the reaction stop solution was added to simultaneously initiate the sea-kidney luciferase luminescence reaction and to read the second set of RLU values. By calculating the ratio of fire *Drosophila* luciferase to sea-kidney luciferase activity in each sample (i.e., the normalized ratio), inter-well errors were effectively eliminated, and the normalized ratio of the *miR-276a* mimic-treated group against the negative control group was assessed to determine whether *miR-276a* can specifically bind to the target site on the 3′ UTR of the *nej* gene and exert post-transcriptional repressive regulation.

#### 4.3.4. Phalloidin Staining Detection

After completing the aforementioned dissection procedures to obtain a semi-intact *Drosophila* heart with a complete morphological structure, the *Drosophila* body fluid was quickly and carefully aspirated from the original Petri dish using a pipette, and immediately replaced with pre-chilled relaxation buffer (this buffer contains 10 mM EGTA to effectively chelate calcium ions, thereby promoting relaxation of the cardiac muscle fibers and inhibiting excessive contraction), a sufficient volume of 4% paraformaldehyde fixative was then added to the sample and fix it at room temperature in the dark for 25 min to ensure thorough cross-linking and stabilization of the tissue protein structure. After fixation, the sample was gently rinsed three times with PBS, with each rinse lasting 10 min, to thoroughly remove residual fixative and restore an appropriate ionic environment. Next, specific staining was performed by directly adding a working solution of the fluorescently labeled phalloidin (phalloidin, a toxin that binds to filamentous F-actin with high affinity and specificity and is commonly used to label the cytoskeleton) directly onto the heart tissue to ensure complete coverage. The entire culture dish was then placed on a variable-speed shaker and incubated at room temperature under strict light-shielding conditions for 40 min at low speed to ensure the dye fully penetrated the tissue and achieved uniform binding; After the staining incubation, the excess phalloidin staining solution was carefully removed, and the sample was washed three times with fresh PBS, with each wash lasting 10 min, to effectively remove unbound nonspecific background staining and thereby reduce fluorescence noise during imaging. For mounting, a small drop of anti-fluorescence quenching mountant was placed in the center of a clean microscope slide. Using a pipette, the stained heart tissue was gently transferred along with a small amount of liquid to the center of this drop. Then, forceps were used to pick up a clean coverslip, first touching one edge to the droplet, then slowly and evenly lowering the other edge, allowing the surface tension of the liquid to naturally adhere the coverslip. We were sure to avoid creating air bubbles or causing the tissue to curl or fold, ensuring the sample remains flat and in the same focal plane. Finally, the prepared slide was immediately placed on the stage of a stereofluorescence microscope (or confocal microscope). We quickly focused at the excitation wavelength corresponding to the fluorescent dye used and captured the image. Given that fluorescent dyes are prone to photobleaching, minimize the exposure time as much as possible and maintain consistent imaging conditions throughout the acquisition process, Finally, we used the acquired fluorescence images to systematically analyze the orientation, density distribution, and overall cytoskeletal structural characteristics of F-actin in the cardiac myofibrils of *Drosophila* hearts.

#### 4.3.5. Climbing Ability Test

The negative-geotaxis climbing ability of *Drosophila* was assessed using a custom-built apparatus, as previously described [60]. The climbing apparatus was separate from the apparatus used for the exercise intervention. It consisted of a transparent glass tube measuring 18 cm in length and 2.8 cm in internal diameter. A soft sponge pad was placed at the bottom of the tube to cushion the *Drosophila* during tapping. A horizontal boundary line was marked 12 cm above the bottom of the tube, corresponding to one-third of the tube length from its upper end.

One day before testing, age-matched healthy *Drosophila* were randomly allocated to groups of 12 and maintained overnight in separate vials at 25 °C and 60% relative humidity under a 12 h light/12 h dark cycle. This procedure allowed the *Drosophila* to acclimate to the testing groups and minimized the potential influence of regrouping-related stress on climbing ability.

On the day of testing, each group of 12 *Drosophila* was gently transferred into a climbing tube, which was then sealed with a cotton plug and maintained vertically. The *Drosophila* were allowed to acclimate to the apparatus for 10 min. At the beginning of each trial, the tube was [manually tapped/mechanically displaced] for approximately 1 s to bring all *Drosophila* to the bottom. The procedure was repeated once every 15 s for a total of 10 trials, allowing the *Drosophila* to restart their negative-geotaxis response after each displacement.

The climbing process was recorded using a camera positioned directly in front of the tube at a fixed distance, focal length, and aperture ([manufacturer and model], [actual frame rate] frames/s). For the fourth, fifth, and sixth trials, video frames were extracted 10 s after the *Drosophila* had been displaced to the bottom. A single investigator, blinded to the experimental groups, counted the number of *Drosophila* located above the 12 cm boundary and the total number of visible *Drosophila* in the tube. When overlapping or obscured *Drosophila* could not be reliably distinguished in a single frame, frame-by-frame video inspection was used to confirm the count.

The indices obtained from the fourth, fifth, and sixth trials were averaged to generate one final climbing index for each group of *Drosophila*. These repeated trials were treated as technical repetitions rather than independent biological replicates. The climbing index was used only as an auxiliary measure of general climbing ability and functional status; it was not interpreted as a cardiac-specific measure of recovery or as evidence of equivalent exercise exposure across genotypes.

### 4.4. Statistical Analysis

Graphs were plotted using GraphPad Prism 9 software, and data were statistically analyzed using Statistical Package for Social Sciences (SPSS) version 25. An independent samples *t*-test was used to compare the W^1118^ and W^1118^ + E groups. One-way analysis of variance (ANOVA) was employed to compare three sets of data. Where data did not conform to a normal distribution, non-parametric Kruskal–Wallis one-way analysis of variance (K-sample) was utilized for analysis. The effects of the exercise intervention were compared using a two-way analysis of variance, with Tukey’s post hoc test employed. All data are expressed as mean ± standard error of the mean (X ± SEM), * *p* < 0.05, ** *p* < 0.01, *** *p* < 0.001.

## 5. Conclusions

Taken together, our findings demonstrate that alterations in *miR-276a* and *nej* expression in cardiac samples are associated with cardiac dysfunction in *Drosophila*, while exercise improves cardiac function and modulates *miR-276a*/*nej* expression in a context-dependent manner. Findings from Cg-Gal4-mediated *miR-276a* manipulation suggest a possible contribution of the fat body.

## Figures and Tables

**Table 1:** One-way ANOVA results for the effects of cardiac-specific *nej* manipulation.

	df	F	*p*-Value	Partial η^2^
*nej*	2	648.778	<0.001	0.995
Heart Rate	2	199.619	<0.001	0.847
Heart Period	2	103.917	<0.001	0.743
Diastolic Intervals	2	90.02	<0.001	0.714
Systolic Intervals	2	23.423	<0.001	0.473
Arrhythmia Index	2	10.839	<0.001	0.394
Diastolic Diameter	2	5.457	0.006	0.231
Systolic Diameter	2	10.761	<0.001	0.23
Fractional Shortening	2	32.794	<0.001	0.137
Climbing Ability	2	53.742	<0.001	0.947

**Table 2:** Post hoc pairwise comparisons among cardiac-specific *nej* genotypes.

	Group1	Group2	*p*-Value	95% CI Lower	95% CI Upper
*nej*	Hand-Gal4 > W^1118^	Hand-Gal4 > UAS-nej^OE^	<0.001	−2.523	−2.011
	Hand-Gal4 > W^1118^	Hand-Gal4 > UAS-nej^RNAi^	0.001	0.317	0.829
	Hand-Gal4 > UAS-nej^OE^	Hand-Gal4 > UAS-nej^RNAi^	0.001	2.584	3.096
Heart Rate	Hand-Gal4 > W^1118^	Hand-Gal4 > UAS-nej^OE^	<0.001	1.180	1.600
	Hand-Gal4 > W^1118^	Hand-Gal4 > UAS-nej^RNAi^	0.001	0.980	1.400
	Hand-Gal4 > UAS-nej^OE^	Hand-Gal4 > UAS-nej^RNAi^	0.001	−0.330	−0.060
Heart Period	Hand-Gal4 > W^1118^	Hand-Gal4 > UAS-nej^OE^	<0.001	−1.450	−0.999
	Hand-Gal4 > W^1118^	Hand-Gal4 > UAS-nej^RNAi^	0.001	−0.899	−0.573
	Hand-Gal4 > UAS-nej^OE^	Hand-Gal4 > UAS-nej^RNAi^	0.001	0.245	0.732
Diastolic Intervals	Hand-Gal4 > W^1118^	Hand-Gal4 > UAS-nej^OE^	<0.001	−1.390	−0.930
	Hand-Gal4 > W^1118^	Hand-Gal4 > UAS-nej^RNAi^	0.001	−0.877	−0.551
	Hand-Gal4 > UAS-nej^OE^	Hand-Gal4 > UAS-nej^RNAi^	0.001	0.195	0.697
Systolic Intervals	Hand-Gal4 > W^1118^	Hand-Gal4 > UAS-nej^OE^	<0.001	−0.093	−0.038
	Hand-Gal4 > W^1118^	Hand-Gal4 > UAS-nej^RNAi^	0.001	−0.044	−0.002
	Hand-Gal4 > UAS-nej^OE^	Hand-Gal4 > UAS-nej^RNAi^	0.001	0.018	0.065
Arrhythmia Index	Hand-Gal4 > W^1118^	Hand-Gal4 > UAS-nej^OE^	0.001	−0.209	−0.050
	Hand-Gal4 > W^1118^	Hand-Gal4 > UAS-nej^RNAi^	0.001	−0.417	−0.089
	Hand-Gal4 > UAS-nej^OE^	Hand-Gal4 > UAS-nej^RNAi^	0.001	−0.276	0.029
Diastolic Diameter	Hand-Gal4 > W^1118^	Hand-Gal4 > UAS-nej^OE^	0.006	−70.884	−9.816
	Hand-Gal4 > W^1118^	Hand-Gal4 > UAS-nej^RNAi^	0.049	−61.155	−0.087
	Hand-Gal4 > UAS-nej^OE^	Hand-Gal4 > UAS-nej^RNAi^	0.727	−20.806	40.263
Systolic Diameter	Hand-Gal4 > W^1118^	Hand-Gal4 > UAS-nej^OE^	<0.001	−77.822	−24.711
	Hand-Gal4 > W^1118^	Hand-Gal4 > UAS-nej^RNAi^	0.025	−56.248	−3.136
	Hand-Gal4 > UAS-nej^OE^	Hand-Gal4 > UAS-nej^RNAi^	0.134	−4.981	48.130
Fractional Shortening	Hand-Gal4 > W^1118^	Hand-Gal4 > UAS-nej^OE^	<0.001	0.084	0.165
	Hand-Gal4 > W^1118^	Hand-Gal4 > UAS-nej^RNAi^	<0.001	0.071	0.152
	Hand-Gal4 > UAS-nej^OE^	Hand-Gal4 > UAS-nej^RNAi^	0.724	−0.054	0.028
Climbing Ability	Hand-Gal4 > W^1118^	Hand-Gal4 > UAS-nej^OE^	<0.001	0.131	0.289
	Hand-Gal4 > W^1118^	Hand-Gal4 > UAS-nej^RNAi^	<0.001	0.168	0.325
	Hand-Gal4 > UAS-nej^OE^	Hand-Gal4 > UAS-nej^RNAi^	0.386	−0.042	0.115

**Table 3:** Two-way ANOVA results for the effects of reporter construct and mimic treatment on relative luciferase activity.

Effect	df	F	*p*-Value	Partial η^2^
reporter construct	1	123.851	<0.001	0.912
mimic treatment	2	86.721	<0.001	0.935
reporter construct × mimic treatment	2	91.418	<0.001	0.938

**Table 4:** Simple-effects comparisons of mimic treatments within each reporter construct and of reporter constructs within each mimic treatment.

Fixed Condition	Group1	Group2	*p*-Value	95% CI Lower	95% CI Upper
Control	mimics NC	mimics	0.234	−0.036	0.133
WT	mimics NC	mimics	<0.001	0.591	0.759
Mut	mimics NC	mimics	0.584	−0.062	0.106
mimics NC	Control	WT	0.971	−0.123	0.091
	Control	Mut	0.981	−0.121	0.094
	WT	Mut	1.000	−0.105	0.109
mimics	Control	WT	<0.001	0.504	0.718
	Control	Mut	0.685	−0.147	0.067
	WT	Mut	<0.001	−0.758	−0.544

**Table 5:** One-way ANOVA results for the effects of cardiac-specific *miR-276a* genotypes.

	df	F	*p*-Value	Partial η^2^
*miR-276a*	2	145.012	<0.001	0.98
*nej*	2	275.731	<0.001	0.989
Heart Rate	2	61.591	<0.001	0.631
Heart Period	2	30.123	<0.001	0.456
Diastolic Intervals	2	24.468	<0.001	0.405
Systolic Intervals	2	8.679	<0.001	0.194
Arrhythmia Index	2	20.599	<0.001	0.364
Diastolic Diameter	2	16.866	<0.001	0.319
Systolic Diameter	2	10.954	<0.001	0.233
Fractional Shortening	2	34.71	<0.001	0.491
Climbing Ability	2	28.529	0.001	0.905

**Table 6:** Post hoc pairwise comparisons among cardiac-specific *miR-276a* genotypes.

	Group1	Group2	*p*-Value	95% CI Lower	95% CI Upper
*miR-276a*	Hand-Gal4 > W^1118^	Hand-Gal4 > UAS-miR-276a^RNAi^	<0.001	0.457	0.936
	Hand-Gal4 > W^1118^	Hand-Gal4 > UAS-miR-276a^OE^	<0.001	−0.870	−0.392
	Hand-Gal4 > UAS-miR-276a^RNAi^	Hand-Gal4 > UAS-miR-276a^OE^	<0.001	−1.567	−1.088
*nej*	Hand > W^1118^	Hand > UAS-miR-276a^RNAi^	<0.001	−1.336	−0.952
	Hand > W^1118^	Hand > UAS-miR-276a^OE^	0.025	0.036	0.420
	Hand > UAS-miR-276a^RNAi^	Hand > UAS-miR-276a^OE^	<0.001	1.180	1.564
Heart Rate	Hand > W^1118^	Hand > UAS-miR-276a^RNAi^	<0.001	0.440	0.730
	Hand > W^1118^	Hand > UAS-miR-276a^OE^	<0.001	0.440	0.730
	Hand > UAS-miR-276a^RNAi^	Hand > UAS-miR-276a^OE^	1.000	−0.150	0.150
Heart Period	Hand > W^1118^	Hand > UAS-miR-276a^RNAi^	<0.001	−0.359	−0.191
	Hand > W^1118^	Hand > UAS-miR-276a^OE^	<0.001	−0.363	−0.159
	Hand > UAS-miR-276a^RNAi^	Hand > UAS-miR-276a^OE^	0.985	−0.096	0.124
Diastolic Intervals	Hand > W^1118^	Hand > UAS-miR-276a^RNAi^	<0.001	−0.345	−0.171
	Hand > W^1118^	Hand > UAS-miR-276a^OE^	<0.001	−0.346	−0.137
	Hand > UAS-miR-276a^RNAi^	Hand > UAS-miR-276a^OE^	0.978	−0.098	0.131
Systolic Intervals	Hand > W^1118^	Hand > UAS-miR-276a^RNAi^	<0.001	−0.054	−0.014
	Hand > W^1118^	Hand > UAS-miR-276a^OE^	0.030	−0.042	−0.002
	Hand > UAS-miR-276a^RNAi^	Hand > UAS-miR-276a^OE^	0.290	−0.007	0.033
Arrhythmia Index	Hand > W^1118^	Hand > UAS-miR-276a^RNAi^	<0.001	−0.309	−0.148
	Hand > W^1118^	Hand > UAS-miR-276a^OE^	<0.001	−0.279	−0.099
	Hand > UAS-miR-276a^RNAi^	Hand > UAS-miR-276a^OE^	0.765	−0.073	0.153
Diastolic Diameter	Hand > W^1118^	Hand > UAS-miR-276a^RNAi^	<0.001	−36.717	−13.698
	Hand > W^1118^	Hand > UAS-miR-276a^OE^	<0.001	−34.535	−11.516
	Hand > UAS-miR-276a^RNAi^	Hand > UAS-miR-276a^OE^	0.893	−9.328	13.691
Systolic Diameter	Hand > W^1118^	Hand > UAS-miR-276a^RNAi^	<0.001	−31.242	−9.782
	Hand > W^1118^	Hand > UAS-miR-276a^OE^	0.007	−24.829	−3.369
	Hand > UAS-miR-276a^RNAi^	Hand > UAS-miR-276a^OE^	0.331	−4.317	17.143
Fractional Shortening	Hand > W^1118^	Hand > UAS-miR-276a^RNAi^	<0.001	0.047	0.094
	Hand > W^1118^	Hand > UAS-miR-276a^OE^	<0.001	0.048	0.095
	Hand > UAS-miR-276a^RNAi^	Hand > UAS-miR-276a^OE^	0.994	−0.023	0.025
Climbing Ability	Hand > W^1118^	Hand > UAS-miR-276a^RNAi^	0.001	0.084	0.199
	Hand > W^1118^	Hand > UAS-miR-276a^OE^	0.017	0.017	0.133
	Hand > UAS-miR-276a^RNAi^	Hand > UAS-miR-276a^OE^	0.028	−0.124	−0.009

**Table 7:** One-way ANOVA results for the effects among experimental groups.

	df	F	*p*-Value	Partial η^2^
*miR-276a* (fat body)	2	813.966	<0.001	0.996
*miR-276a* (heart)	2	118.775	<0.001	0.975
*nej*	2	79.96	<0.001	0.964
Heart Rate	2	16.68	<0.001	0.317
Heart Period	2	8.895	<0.001	0.198
Diastolic Intervals	2	4.67	0.012	0.115
Systolic Intervals	2	7.775	0.001	0.178
Arrhythmia Index	2	7.164	0.001	0.168
Diastolic Diameter	2	13.545	<0.001	0.273
Systolic Diameter	2	6.869	0.002	0.16
Fractional Shortening	2	11.914	<0.001	0.249
Climbing Ability	2	13.77	0.006	0.821

**Table 8:** Post hoc pairwise comparisons among experimental groups.

	Group1	Group2	*p*-Value	95% CI Lower	95% CI Upper
*miR-276a* (fat body)	Cg-Gal4 > W^1118^	Cg-Gal4 > UAS-miR-276a^RNAi^	<0.001	0.586	1.030
	Cg-Gal4 > W^1118^	Cg-Gal4 > UAS-miR-276a^OE^	<0.001	−2.248	−1.804
	Cg-Gal4 > UAS-miR-276a^RNAi^	Cg-Gal4 > UAS-miR-276a^OE^	<0.001	−3.056	−2.612
*miR-276a* (heart)	Cg-Gal4 > W^1118^	Cg-Gal4 > UAS-miR-276a^RNAi^	0.003	0.312	1.074
	Cg-Gal4 > W^1118^	Cg-Gal4 > UAS-miR-276a^OE^	<0.001	−1.579	−0.817
	Cg-Gal4 > UAS-miR-276a^RNAi^	Cg-Gal4 > UAS-miR-276a^OE^	<0.001	−2.272	−1.510
*nej*	Cg-Gal4 > W^1118^	Cg-Gal4 > UAS-miR-276a^RNAi^	0.025	−0.534	−0.045
	Cg-Gal4 > W^1118^	Cg-Gal4 > UAS-miR-276a^OE^	<0.001	0.447	0.936
	Cg-Gal4 > UAS-miR-276a^RNAi^	Cg-Gal4 > UAS-miR-276a^OE^	<0.001	0.736	1.226
Heart Rate	Cg-Gal4 > W^1118^	Cg-Gal4 > UAS-miR-276a^RNAi^	<0.001	0.210	0.510
	Cg-Gal4 > W^1118^	Cg-Gal4 > UAS-miR-276a^OE^	0.019	0.020	0.320
	Cg-Gal4 > UAS-miR-276a^RNAi^	Cg-Gal4 > UAS-miR-276a^OE^	0.011	−0.330	−0.040
Heart Period	Cg-Gal4 > W^1118^	Cg-Gal4 > UAS-miR-276a^RNAi^	<0.001	−0.178	−0.047
	Cg-Gal4 > W^1118^	Cg-Gal4 > UAS-miR-276a^OE^	0.382	−0.102	0.029
	Cg-Gal4 > UAS-miR-276a^RNAi^	Cg-Gal4 > UAS-miR-276a^OE^	0.018	0.011	0.142
Diastolic Intervals	Cg-Gal4 > W^1118^	Cg-Gal4 > UAS-miR-276a^RNAi^	0.009	−0.154	−0.019
	Cg-Gal4 > W^1118^	Cg-Gal4 > UAS-miR-276a^OE^	0.305	−0.109	0.026
	Cg-Gal4 > UAS-miR-276a^RNAi^	Cg-Gal4 > UAS-miR-276a^OE^	0.264	−0.023	0.112
Systolic Intervals	Cg-Gal4 > W^1118^	Cg-Gal4 > UAS-miR-276a^RNAi^	0.029	−0.046	−0.002
	Cg-Gal4 > W^1118^	Cg-Gal4 > UAS-miR-276a^OE^	0.426	−0.010	0.033
	Cg-Gal4 > UAS-miR-276a^RNAi^	Cg-Gal4 > UAS-miR-276a^OE^	0.001	0.014	0.057
Arrhythmia Index	Cg-Gal4 > W^1118^	Cg-Gal4 > UAS-miR-276a^RNAi^	0.014	−0.080	−0.007
	Cg-Gal4 > W^1118^	Cg-Gal4 > UAS-miR-276a^OE^	0.723	−0.028	0.013
	Cg-Gal4 > UAS-miR-276a^RNAi^	Cg-Gal4 > UAS-miR-276a^OE^	0.040	0.001	0.071
Diastolic Diameter	Cg-Gal4 > W^1118^	Cg-Gal4 > UAS-miR-276a^RNAi^	<0.001	−38.300	−13.525
	Cg-Gal4 > W^1118^	Cg-Gal4 > UAS-miR-276a^OE^	0.001	−31.729	−6.954
	Cg-Gal4 > UAS-miR-276a^RNAi^	Cg-Gal4 > UAS-miR-276a^OE^	0.417	−5.816	18.958
Systolic Diameter	Cg-Gal4 > W^1118^	Cg-Gal4 > UAS-miR-276a^RNAi^	0.001	−23.572	−4.964
	Cg-Gal4 > W^1118^	Cg-Gal4 > UAS-miR-276a^OE^	0.064	−18.187	0.421
	Cg-Gal4 > UAS-miR-276a^RNAi^	Cg-Gal4 > UAS-miR-276a^OE^	0.354	−3.919	14.689
Fractional Shortening	Cg-Gal4 > W^1118^	Cg-Gal4 > UAS-miR-276a^RNAi^	<0.001	0.029	0.086
	Cg-Gal4 > W^1118^	Cg-Gal4 > UAS-miR-276a^OE^	0.013	0.006	0.063
	Cg-Gal4 > UAS-miR-276a^RNAi^	Cg-Gal4 > UAS-miR-276a^OE^	0.139	−0.051	0.006
Climbing Ability	Cg-Gal4 > W^1118^	Cg-Gal4 > UAS-miR-276a^RNAi^	0.005	0.091	0.365
	Cg-Gal4 > W^1118^	Cg-Gal4 > UAS-miR-276a^OE^	0.361	−0.071	0.204
	Cg-Gal4 > UAS-miR-276a^RNAi^	Cg-Gal4 > UAS-miR-276a^OE^	0.026	−0.299	−0.024

**Table 9:** Simple-effects analyses of exercise intervention and genotype.

	Fixed Condition	Group1	Group2	*p*-Value	95% CI Lower	95% CI Upper
*miR-276a*		NC	NE	<0.001	−1.925	−1.608
*nej*		NC	NE	0.005	0.128	0.394
*miR-276a*	Control	Hand > W^1118^	Hand > UAS-miR-276a^RNAi^	<0.001	0.497	0.773
		Hand > W^1118^	Hand > UAS-miR-276a^OE^	<0.001	−0.473	−0.197
		Hand > UAS-miR-276a^RNAi^	Hand > UAS-miR-276a^OE^	<0.001	−1.108	−0.832
	Exercise	Hand > W^1118^	Hand > UAS-miR-276a^RNAi^	<0.001	1.895	2.171
		Hand > W^1118^	Hand > UAS-miR-276a^OE^	<0.001	1.252	1.528
		Hand > UAS-miR-276a^RNAi^	Hand > UAS-miR-276a^OE^	<0.001	−0.781	−0.505
	Hand > W^1118^	Control	Exercise	<0.001	−1.874	−1.657
	Hand > UAS-miR-276a^RNAi^	Control	Exercise	<0.001	−0.475	−0.259
	Hand > UAS-miR-276a^OE^	Control	Exercise	0.437	−0.148	0.068
*nej*	Control	Hand > W^1118^	Hand > UAS-miR-276a^RNAi^	<0.001	−0.592	−0.279
		Hand > W^1118^	Hand > UAS-miR-276a^OE^	<0.001	0.681	0.994
		Hand > UAS-miR-276a^RNAi^	Hand > UAS-miR-276a^OE^	<0.001	0.683	0.992
	Exercise	Hand > W^1118^	Hand > UAS-miR-276a^RNAi^	<0.001	−0.478	−0.165
		Hand > W^1118^	Hand > UAS-miR-276a^OE^	0.003	0.085	0.398
		Hand > UAS-miR-276a^RNAi^	Hand > UAS-miR-276a^OE^	<0.001	0.406	0.719
	Hand > W^1118^	Control	Exercise	0.001	0.137	0.382
	Hand > UAS-miR-276a^RNAi^	Control	Exercise	<0.001	0.251	0.496
	Hand > UAS-miR-276a^OE^	Control	Exercise	0.106	−0.221	0.024
Heart Rate	Control	Hand-Gal4 > W^1118^	Hand-Gal4 > UAS-miR-276a^RNAi^	<0.001	0.468	0.705
		Hand-Gal4 > W^1118^	Hand-Gal4 > UAS-miR-276a^OE^	<0.001	0.469	0.706
		Hand-Gal4 > UAS-miR-276a^RNAi^	Hand-Gal4 > UAS-miR-276a^OE^	1	−0.118	0.119
	Exercise	Hand-Gal4 > W^1118^	Hand-Gal4 > UAS-miR-276a^RNAi^	<0.001	0.274	0.511
		Hand-Gal4 > W^1118^	Hand-Gal4 > UAS-miR-276a^OE^	<0.001	0.327	0.564
		Hand-Gal4 > UAS-miR-276a^RNAi^	Hand-Gal4 > UAS-miR-276a^OE^	0.851	−0.066	0.171
	Hand-Gal4 > W^1118^	Control	Exercise	0.698	−0.116	0.078
	Hand-Gal4 > UAS-miR-276a^RNAi^	Control	Exercise	<0.001	−0.310	−0.116
	Hand-Gal4 > UAS-miR-276a^OE^	Control	Exercise	0.001	−0.258	−0.064
Heart Period	Control	Hand-Gal4 > W^1118^	Hand-Gal4 > UAS-miR-276a^RNAi^	<0.001	−0.350	−0.200
		Hand-Gal4 > W^1118^	Hand-Gal4 > UAS-miR-276a^OE^	<0.001	−0.336	−0.186
		Hand-Gal4 > UAS-miR-276a^RNAi^	Hand-Gal4 > UAS-miR-276a^OE^	1	−0.061	0.089
	Exercise	Hand-Gal4 > W^1118^	Hand-Gal4 > UAS-miR-276a^RNAi^	<0.001	−0.212	−0.063
		Hand-Gal4 > W^1118^	Hand-Gal4 > UAS-miR-276a^OE^	<0.001	−0.233	−0.083
		Hand-Gal4 > UAS-miR-276a^RNAi^	Hand-Gal4 > UAS-miR-276a^OE^	1	−0.095	0.054
	Hand-Gal4 > W^1118^	Control	Exercise	0.388	−0.034	0.088
	Hand-Gal4 > UAS-miR-276a^RNAi^	Control	Exercise	<0.001	0.103	0.226
	Hand-Gal4 > UAS-miR-276a^OE^	Control	Exercise	<0.001	0.069	0.191
Diastolic Intervals	Control	Hand-Gal4 > W^1118^	Hand-Gal4 > UAS-miR-276a^RNAi^	<0.001	−0.335	−0.181
		Hand-Gal4 > W^1118^	Hand-Gal4 > UAS-miR-276a^OE^	<0.001	−0.318	−0.165
		Hand-Gal4 > UAS-miR-276a^RNAi^	Hand-Gal4 > UAS-miR-276a^OE^	1	−0.060	0.093
	Exercise	Hand-Gal4 > W^1118^	Hand-Gal4 > UAS-miR-276a^RNAi^	<0.001	−0.214	−0.060
		Hand-Gal4 > W^1118^	Hand-Gal4 > UAS-miR-276a^OE^	<0.001	−0.227	−0.074
		Hand-Gal4 > UAS-miR-276a^RNAi^	Hand-Gal4 > UAS-miR-276a^OE^	1	−0.090	0.063
	Hand-Gal4 > W^1118^	Control	Exercise	0.159	−0.018	0.107
	Hand-Gal4 > UAS-miR-276a^RNAi^	Control	Exercise	<0.001	0.103	0.228
	Hand-Gal4 > UAS-miR-276a^OE^	Control	Exercise	<0.001	0.073	0.198
Systolic Intervals	Control	Hand-Gal4 > W^1118^	Hand-Gal4 > UAS-miR-276a^RNAi^	<0.001	−0.050	−0.018
		Hand-Gal4 > W^1118^	Hand-Gal4 > UAS-miR-276a^OE^	0.004	−0.038	−0.006
		Hand-Gal4 > UAS-miR-276a^RNAi^	Hand-Gal4 > UAS-miR-276a^OE^	0.181	−0.004	0.029
	Exercise	Hand-Gal4 > W^1118^	Hand-Gal4 > UAS-miR-276a^RNAi^	0.381	−0.026	0.006
		Hand-Gal4 > W^1118^	Hand-Gal4 > UAS-miR-276a^OE^	0.146	−0.029	0.003
		Hand-Gal4 > UAS-miR-276a^RNAi^	Hand-Gal4 > UAS-miR-276a^OE^	1	−0.019	0.013
	Hand-Gal4 > W^1118^	Control	Exercise	0.201	−0.005	0.022
	Hand-Gal4 > UAS-miR-276a^RNAi^	Control	Exercise	<0.001	0.019	0.046
	Hand-Gal4 > UAS-miR-276a^OE^	Control	Exercise	0.012	0.004	0.030
Arrhythmia index	Control	Hand-Gal4 > W^1118^	Hand-Gal4 > UAS-miR-276a^RNAi^	<0.001	−0.309	−0.148
		Hand-Gal4 > W^1118^	Hand-Gal4 > UAS-miR-276a^OE^	<0.001	−0.269	−0.108
		Hand-Gal4 > UAS-miR-276a^RNAi^	Hand-Gal4 > UAS-miR-276a^OE^	0.687	−0.040	0.120
	Exercise	Hand-Gal4 > W^1118^	Hand-Gal4 > UAS-miR-276a^RNAi^	1	−0.061	0.100
		Hand-Gal4 > W^1118^	Hand-Gal4 > UAS-miR-276a^OE^	0.057	−0.159	0.002
		Hand-Gal4 > UAS-miR-276a^RNAi^	Hand-Gal4 > UAS-miR-276a^OE^	0.011	−0.178	−0.018
	Hand-Gal4 > W^1118^	Control	Exercise	0.498	−0.043	0.088
	Hand-Gal4 > UAS-miR-276a^RNAi^	Control	Exercise	<0.001	0.205	0.336
	Hand-Gal4 > UAS-miR-276a^OE^	Control	Exercise	<0.001	0.067	0.198
Diastolic diameter	Control	Hand-Gal4 > W^1118^	Hand-Gal4 > UAS-miR-276a^RNAi^	<0.001	−35.329	−15.086
		Hand-Gal4 > W^1118^	Hand-Gal4 > UAS-miR-276a^OE^	<0.001	−33.147	−12.904
		Hand-Gal4 > UAS-miR-276a^RNAi^	Hand-Gal4 > UAS-miR-276a^OE^	1	−7.940	12.303
	Exercise	Hand-Gal4 > W^1118^	Hand-Gal4 > UAS-miR-276a^RNAi^	<0.001	−31.848	−11.605
		Hand-Gal4 > W^1118^	Hand-Gal4 > UAS-miR-276a^OE^	0.019	−21.700	−1.457
		Hand-Gal4 > UAS-miR-276a^RNAi^	Hand-Gal4 > UAS-miR-276a^OE^	0.049	0.026	20.270
	Hand-Gal4 > W^1118^	Control	Exercise	<0.001	9.318	25.837
	Hand-Gal4 > UAS-miR-276a^RNAi^	Control	Exercise	<0.001	12.799	29.318
	Hand-Gal4 > UAS-miR-276a^OE^	Control	Exercise	<0.001	20.766	37.284
Systolic diameter	Control	Hand-Gal4 > W^1118^	Hand-Gal4 > UAS-miR-276a^RNAi^	<0.001	−29.674	−11.351
		Hand-Gal4 > W^1118^	Hand-Gal4 > UAS-miR-276a^OE^	0.001	−23.261	−4.937
		Hand-Gal4 > UAS-miR-276a^RNAi^	Hand-Gal4 > UAS-miR-276a^OE^	0.276	−2.749	15.575
	Exercise	Hand-Gal4 > W^1118^	Hand-Gal4 > UAS-miR-276a^RNAi^	0.133	−16.835	1.488
		Hand-Gal4 > W^1118^	Hand-Gal4 > UAS-miR-276a^OE^	<0.001	−26.601	−8.278
		Hand-Gal4 > UAS-miR-276a^RNAi^	Hand-Gal4 > UAS-miR-276a^OE^	0.032	−18.928	−0.604
	Hand-Gal4 > W^1118^	Control	Exercise	<0.001	11.687	26.639
	Hand-Gal4 > UAS-miR-276a^RNAi^	Control	Exercise	<0.001	24.526	39.478
	Hand-Gal4 > UAS-miR-276a^OE^	Control	Exercise	<0.001	8.347	23.299
Fractional shortening	Control	Hand-Gal4 > W^1118^	Hand-Gal4 > UAS-miR-276a^RNAi^	<0.001	0.034	0.107
		Hand-Gal4 > W^1118^	Hand-Gal4 > UAS-miR-276a^OE^	<0.001	0.035	0.108
		Hand-Gal4 > UAS-miR-276a^RNAi^	Hand-Gal4 > UAS-miR-276a^OE^	1.000	−0.035	0.037
	Exercise	Hand-Gal4 > W^1118^	Hand-Gal4 > UAS-miR-276a^RNAi^	0.568	−0.017	0.056
		Hand-Gal4 > W^1118^	Hand-Gal4 > UAS-miR-276a^OE^	0.471	−0.015	0.058
		Hand-Gal4 > UAS-miR-276a^RNAi^	Hand-Gal4 > UAS-miR-276a^OE^	1.000	−0.035	0.038
	Hand-Gal4 > W^1118^	Control	Exercise	0.090	−0.055	0.004
	Hand-Gal4 > UAS-miR-276a^RNAi^	Control	Exercise	<0.001	−0.106	−0.047
	Hand-Gal4 > UAS-miR-276a^OE^	Control	Exercise	<0.001	−0.106	−0.046
Climbing Capacity	Control	Hand-Gal4 > W^1118^	Hand-Gal4 > UAS-miR-276a^RNAi^	<0.001	0.095	0.188
		Hand-Gal4 > W^1118^	Hand-Gal4 > UAS-miR-276a^OE^	0.002	0.028	0.122
		Hand-Gal4 > UAS-miR-276a^RNAi^	Hand-Gal4 > UAS-miR-276a^OE^	0.006	−0.113	−0.020
	Exercise	Hand-Gal4 > W^1118^	Hand-Gal4 > UAS-miR-276a^RNAi^	<0.001	0.107	0.200
		Hand-Gal4 > W^1118^	Hand-Gal4 > UAS-miR-276a^OE^	<0.001	0.067	0.160
		Hand-Gal4 > UAS-miR-276a^RNAi^	Hand-Gal4 > UAS-miR-276a^OE^	0.105	−0.087	0.007
	Hand-Gal4 > W^1118^	Control	Exercise	<0.001	−0.136	−0.063
	Hand-Gal4 > UAS-miR-276a^RNAi^	Control	Exercise	<0.001	−0.124	−0.051
	Hand-Gal4 > UAS-miR-276a^OE^	Control	Exercise	0.003	−0.098	−0.024

**Table 10:** Two-way ANOVA results for the effects of genotype and exercise intervention.

	Effect	df	F	*p*-Value	Partial η^2^
*miR-276a*	Genotype	2	730.527	<0.001	0.992
	Exercise	1	636.538	<0.001	0.981
	Genotype × Exercise	2	339.923	<0.001	0.983
*nej*	Genotype	2	154.967	<0.001	0.963
	Exercise	1	56.207	<0.001	0.824
	Genotype × Exercise	2	6.018	0.015	0.501
Heart Rate	Genotype	2	141.035	<0.001	0.662
	Exercise	1	21.494	<0.001	0.13
	Genotype × Exercise	2	4.207	0.017	0.055
Heart Period	Genotype	2	60.342	<0.001	0.456
	Exercise	1	36.007	<0.001	0.2
	Genotype × Exercise	2	5.377	0.006	0.069
Diastolic Intervals	Genotype	2	51.5	<0.001	0.417
	Exercise	1	39.823	<0.001	0.217
	Genotype × Exercise	2	3.945	0.021	0.052
Systolic Intervals	Genotype	2	12.372	<0.001	0.147
	Exercise	1	25.402	<0.001	0.15
	Genotype × Exercise	2	3.36	0.037	0.045
Arrhythmia Index	Genotype	2	17.952	<0.001	0.2
	Exercise	1	54.857	<0.001	0.276
	Genotype × Exercise	2	14.038	<0.001	0.163
Diastolic Diameter	Genotype	2	33.908	<0.001	0.32
	Exercise	1	87.398	<0.001	0.378
	Genotype × Exercise	2	1.977	0.143	0.028
Systolic Diameter	Genotype	2	20.978	<0.001	0.223
	Exercise	1	104.556	<0.001	0.422
	Genotype × Exercise	2	5.11	0.007	0.067
Fractional Shortening	Genotype	2	12.444	<0.001	0.147
	Exercise	1	46.822	<0.001	0.245
	Genotype × Exercise	2	3.778	0.025	0.05
Climbing Ability	Genotype	2	78.714	<0.001	0.929
	Exercise	1	72.573	<0.001	0.858
	Genotype × Exercise	2	1.365	0.292	0.185

**Table 11:** Effects of genotype and exercise intervention in Cg-Gal4/UAS *miR-276a* transgenic *Drosophila*: two-way ANOVA results.

	Effect	df	F	*p*-Value	Partial η^2^
*miR-276a* (fat body)	Genotype	1	426.029	<0.001	0.982
	Exercise	1	225.274	<0.001	0.966
	Genotype × Exercise	1	12.457	0.008	0.609
*miR-276a* (heart)	Genotype	1	13,442.342	<0.001	0.994
	Exercise	1	787.212	<0.001	0.99
	Genotype × Exercise	1	337.708	<0.001	0.977
*nej*	Genotype	1	10.299	0.012	0.563
	Exercise	1	41.528	<0.001	0.838
	Genotype × Exercise	1	3.551	0.096	0.307
Heart Rate	Genotype	2	6.187	0.002	0.052
	Exercise	1	55.792	<0.001	0.199
	Genotype × Exercise	2	7.302	0.001	0.081
Heart Period	Genotype	2	17.688	<0.001	0.137
	Exercise	1	28.771	<0.001	0.114
	Genotype × Exercise	2	0.248	0.781	0.002
Diastolic Intervals	Genotype	2	15.544	<0.001	0.122
	Exercise	1	58.763	<0.001	0.209
	Genotype × Exercise	2	0.165	0.848	0.001
Systolic Intervals	Genotype	2	22.199	<0.001	0.199
	Exercise	1	31.756	<0.001	0.151
	Genotype × Exercise	2	2.645	0.074	0.029
Arrhythmia Index	Genotype	2	17.642	<0.001	0.163
	Exercise	1	265.181	<0.001	0.594
	Genotype × Exercise	2	4.269	0.015	0.045
Diastolic Diameter	Genotype	2	5.118	0.007	0.044
	Exercise	1	15.442	<0.001	0.064
	Genotype × Exercise	2	4.177	0.017	0.036
Systolic Diameter	Genotype	2	7.357	0.001	0.072
	Exercise	1	13.718	<0.001	0.067
	Genotype × Exercise	2	0.390	0.678	0.004
Fractional Shortening	Genotype	2	3.055	0.049	0.029
	Exercise	1	18.747	<0.001	0.084
	Genotype × Exercise	2	1.508	0.224	0.015
Climbing Ability	Genotype	2	25.755	<0.001	0.811
	Exercise	1	152.583	<0.001	0.927
	Genotype × Exercise	2	4.199	0.041	0.412

**Table 12:** Simple-effects pairwise comparisons of gene expression, cardiac function, and climbing ability in Cg-Gal4/UAS *miR-276a* transgenic *Drosophila*.

	Fixed Condition	Group1	Group2	*p*-Value	95% CI Lower	95% CI Upper
*miR-276a* (fat body)	Control	Cg-Gal4 > W^1118^	Cg-Gal4 > UAS-miR-276a^RNAi^	<0.001	0.630	0.926
	Exercise	Cg-Gal4 > W^1118^	Cg-Gal4 > UAS-miR-276a^RNAi^	<0.001	0.951	1.247
	Cg-Gal4 > W^1118^	Control	Exercise	<0.001	−0.991	−0.695
	Cg-Gal4 > UAS-miR-276a^RNAi^	Control	Exercise	<0.001	−0.670	−0.374
*miR-276a* (heart)	Control	Cg-Gal4 > W^1118^	Cg-Gal4 > UAS-miR-276a^RNAi^	<0.001	0.570	0.818
	Exercise	Cg-Gal4 > W^1118^	Cg-Gal4 > UAS-miR-276a^RNAi^	<0.001	1.966	2.214
	Cg-Gal4 > W^1118^	Control	Exercise	<0.001	−1.888	−1.640
	Cg-Gal4 > UAS-miR-276a^RNAi^	Control	Exercise	<0.001	−0.492	−0.244
*nej*	Control	Cg-Gal4 > W^1118^	Cg-Gal4 > UAS-miR-276a^RNAi^	0.007	−0.475	−0.104
	Exercise	Cg-Gal4 > W^1118^	Cg-Gal4 > UAS-miR-276a^RNAi^	0.376	−0.261	0.110
	Cg-Gal4 > W^1118^	Control	Exercise	0.012	0.074	0.445
	Cg-Gal4 > UAS-miR-276a^RNAi^	Control	Exercise	<0.001	0.288	0.659
Heart Rate	Control	Cg-Gal4 > W^1118^	Cg-Gal4 > UAS-miR-276a^RNAi^	<0.001	0.131	0.482
		Cg-Gal4 > W^1118^	Cg-Gal4 > UAS-miR-276a^OE^	1.000	−0.154	0.174
		Cg-Gal4 > UAS-miR-276a^RNAi^	Cg-Gal4 > UAS-miR-276a^OE^	<0.001	−0.466	−0.127
	Exercise	Cg-Gal4 > W^1118^	Cg-Gal4 > UAS-miR-276a^RNAi^	1.000	−0.181	0.110
		Cg-Gal4 > W^1118^	Cg-Gal4 > UAS-miR-276a^OE^	1.000	−0.196	0.097
		Cg-Gal4 > UAS-miR-276a^RNAi^	Cg-Gal4 > UAS-miR-276a^OE^	1.000	−0.169	0.141
	Cg-Gal4 > W^1118^	Control	Exercise	0.018	−0.277	−0.026
	Cg-Gal4 > UAS-miR-276a^RNAi^	Control	Exercise	<0.001	0.356	0.631
	Cg-Gal4 > UAS-miR-276a^OE^	Control	Exercise	0.001	0.083	0.339
Heart Period	Control	Cg-Gal4 > W^1118^	Cg-Gal4 > UAS-miR-276a^RNAi^	0.001	−0.164	−0.036
		Cg-Gal4 > W^1118^	Cg-Gal4 > UAS-miR-276a^OE^	1.000	−0.073	0.048
		Cg-Gal4 > UAS-miR-276a^RNAi^	Cg-Gal4 > UAS-miR-276a^OE^	0.003	0.025	0.150
	Exercise	Cg-Gal4 > W^1118^	Cg-Gal4 > UAS-miR-276a^RNAi^	0.001	−0.136	−0.029
		Cg-Gal4 > W^1118^	Cg-Gal4 > UAS-miR-276a^OE^	1.000	−0.044	0.064
		Cg-Gal4 > UAS-miR-276a^RNAi^	Cg-Gal4 > UAS-miR-276a^OE^	<0.001	0.036	0.149
	Cg-Gal4 > W^1118^	Control	Exercise	0.009	0.016	0.108
	Cg-Gal4 > UAS-miR-276a^RNAi^	Control	Exercise	0.002	0.029	0.130
	Cg-Gal4 > UAS-miR-276a^OE^	Control	Exercise	0.001	0.037	0.132
Diastolic Intervals	Control	Cg-Gal4 > W^1118^	Cg-Gal4 > UAS-miR-276a^RNAi^	0.003	−0.138	−0.023
		Cg-Gal4 > W^1118^	Cg-Gal4 > UAS-miR-276a^OE^	1.000	−0.073	0.035
		Cg-Gal4 > UAS-miR-276a^RNAi^	Cg-Gal4 > UAS-miR-276a^OE^	0.025	0.006	0.118
	Exercise	Cg-Gal4 > W^1118^	Cg-Gal4 > UAS-miR-276a^RNAi^	<0.001	−0.129	−0.034
		Cg-Gal4 > W^1118^	Cg-Gal4 > UAS-miR-276a^OE^	1.000	−0.052	0.044
		Cg-Gal4 > UAS-miR-276a^RNAi^	Cg-Gal4 > UAS-miR-276a^OE^	0.001	0.027	0.128
	Cg-Gal4 > W^1118^	Control	Exercise	<0.001	0.050	0.133
	Cg-Gal4 > UAS-miR-276a^RNAi^	Control	Exercise	<0.001	0.046	0.136
	Cg-Gal4 > UAS-miR-276a^OE^	Control	Exercise	<0.001	0.064	0.149
Systolic Intervals	Control	Cg-Gal4 > W^1118^	Cg-Gal4 > UAS-miR-276a^RNAi^	0.005	−0.035	−0.005
		Cg-Gal4 > W^1118^	Cg-Gal4 > UAS-miR-276a^OE^	0.721	−0.007	0.021
		Cg-Gal4 > UAS-miR-276a^RNAi^	Cg-Gal4 > UAS-miR-276a^OE^	<0.001	0.012	0.042
	Exercise	Cg-Gal4 > W^1118^	Cg-Gal4 > UAS-miR-276a^RNAi^	0.501	−0.027	0.007
		Cg-Gal4 > W^1118^	Cg-Gal4 > UAS-miR-276a^OE^	<0.001	0.012	0.041
		Cg-Gal4 > UAS-miR-276a^RNAi^	Cg-Gal4 > UAS-miR-276a^OE^	<0.001	0.018	0.054
	Cg-Gal4 > W^1118^	Control	Exercise	0.057	0.000	0.023
	Cg-Gal4 > UAS-miR-276a^RNAi^	Control	Exercise	0.004	0.007	0.036
	Cg-Gal4 > UAS-miR-276a^OE^	Control	Exercise	<0.001	0.018	0.042
Arrhythmia Index	Control	Cg-Gal4 > W^1118^	Cg-Gal4 > UAS-miR-276a^RNAi^	0.003	−0.066	−0.011
		Cg-Gal4 > W^1118^	Cg-Gal4 > UAS-miR-276a^OE^	0.009	0.006	0.058
		Cg-Gal4 > UAS-miR-276a^RNAi^	Cg-Gal4 > UAS-miR-276a^OE^	<0.001	0.044	0.098
	Exercise	Cg-Gal4 > W^1118^	Cg-Gal4 > UAS-miR-276a^RNAi^	0.047	−0.058	0.000
		Cg-Gal4 > W^1118^	Cg-Gal4 > UAS-miR-276a^OE^	1.000	−0.030	0.024
		Cg-Gal4 > UAS-miR-276a^RNAi^	Cg-Gal4 > UAS-miR-276a^OE^	0.096	−0.003	0.056
	Cg-Gal4 > W^1118^	Control	Exercise	<0.001	0.094	0.138
	Cg-Gal4 > UAS-miR-276a^RNAi^	Control	Exercise	<0.001	0.101	0.150
	Cg-Gal4 > UAS-miR-276a^OE^	Control	Exercise	<0.001	0.060	0.103
Diastolic Diameter	Control	Cg-Gal4 > W^1118^	Cg-Gal4 > UAS-miR-276a^RNAi^	0.015	−27.238	−2.172
		Cg-Gal4 > W^1118^	Cg-Gal4 > UAS-miR-276a^OE^	0.471	−20.524	5.311
		Cg-Gal4 > UAS-miR-276a^RNAi^	Cg-Gal4 > UAS-miR-276a^OE^	0.510	−5.343	19.540
	Exercise	Cg-Gal4 > W^1118^	Cg-Gal4 > UAS-miR-276a^RNAi^	1.000	−13.987	11.219
		Cg-Gal4 > W^1118^	Cg-Gal4 > UAS-miR-276a^OE^	0.026	1.226	26.733
		Cg-Gal4 > UAS-miR-276a^RNAi^	Cg-Gal4 > UAS-miR-276a^OE^	0.009	3.021	27.706
	Cg-Gal4 > W^1118^	Control	Exercise	0.968	−10.410	10.842
	Cg-Gal4 > UAS-miR-276a^RNAi^	Control	Exercise	0.008	3.640	23.435
	Cg-Gal4 > UAS-miR-276a^OE^	Control	Exercise	<0.001	11.456	32.148
Systolic Diameter	Control	Cg-Gal4 > W^1118^	Cg-Gal4 > UAS-miR-276a^RNAi^	0.001	−20.624	−4.020
		Cg-Gal4 > W^1118^	Cg-Gal4 > UAS-miR-276a^OE^	0.629	−13.220	4.163
		Cg-Gal4 > UAS-miR-276a^RNAi^	Cg-Gal4 > UAS-miR-276a^OE^	0.070	−0.445	16.033
	Exercise	Cg-Gal4 > W^1118^	Cg-Gal4 > UAS-miR-276a^RNAi^	0.189	−17.563	2.234
		Cg-Gal4 > W^1118^	Cg-Gal4 > UAS-miR-276a^OE^	1.000	−13.826	8.655
		Cg-Gal4 > UAS-miR-276a^RNAi^	Cg-Gal4 > UAS-miR-276a^OE^	0.727	−5.383	15.542
	Cg-Gal4 > W^1118^	Control	Exercise	0.125	−1.744	14.236
	Cg-Gal4 > UAS-miR-276a^RNAi^	Control	Exercise	0.002	4.014	17.794
	Cg-Gal4 > UAS-miR-276a^OE^	Control	Exercise	0.056	−0.226	16.604
Fractional Shortening	Control	Cg-Gal4 > W^1118^	Cg-Gal4 > UAS-miR-276a^RNAi^	0.022	0.004	0.074
		Cg-Gal4 > W^1118^	Cg-Gal4 > UAS-miR-276a^OE^	0.852	−0.018	0.047
		Cg-Gal4 > UAS-miR-276a^RNAi^	Cg-Gal4 > UAS-miR-276a^OE^	0.250	−0.058	0.009
	Exercise	Cg-Gal4 > W^1118^	Cg-Gal4 > UAS-miR-276a^RNAi^	1.000	−0.024	0.041
		Cg-Gal4 > W^1118^	Cg-Gal4 > UAS-miR-276a^OE^	0.810	−0.017	0.045
		Cg-Gal4 > UAS-miR-276a^RNAi^	Cg-Gal4 > UAS-miR-276a^OE^	1.000	−0.028	0.040
	Cg-Gal4 > W^1118^	Control	Exercise	0.066	−0.049	0.002
	Cg-Gal4 > UAS-miR-276a^RNAi^	Control	Exercise	<0.001	−0.084	−0.025
	Cg-Gal4 > UAS-miR-276a^OE^	Control	Exercise	0.069	−0.051	0.002
Climbing Ability	Control	Cg-Gal4 > W^1118^	Cg-Gal4 > UAS-mir-276a^RNAi^	<0.001	0.136	0.320
		Cg-Gal4 > W^1118^	Cg-Gal4 > UAS-mir-276a^OE^	0.201	−0.025	0.158
		Cg-Gal4 > UAS-mir-276a^RNAi^	Cg-Gal4 > UAS-mir-276a^OE^	0.001	−0.253	−0.070
	Exercise	Cg-Gal4 > W^1118^	Cg-Gal4 > UAS-mir-276a^RNAi^	0.031	0.008	0.192
		Cg-Gal4 > W^1118^	Cg-Gal4 > UAS-mir-276a^OE^	0.745	−0.052	0.132
		Cg-Gal4 > UAS-mir-276a^RNAi^	Cg-Gal4 > UAS-mir-276a^OE^	0.282	−0.152	0.032
	Cg-Gal4 > W^1118^	Control	Exercise	<0.001	−0.256	−0.112
	Cg-Gal4 > UAS-mir-276a^RNAi^	Control	Exercise	<0.001	−0.384	−0.240
	Cg-Gal4 > UAS-mir-276a^OE^	Control	Exercise	<0.001	0.138	0.282

**Table 13:** *Drosophila* strains.

Strain Number	Abbreviation	Function
BS3605	W^1118^	Wild-type
BS7011	Cg-Gal4	Gal4 transgenic line; Cg is specifically expressed in the fat body
BS33832	Hand-Gal4	Gal4 transgenic line; Hand is specifically expressed in cardiomyocytes
BS61406	UAS-mir-276a^RNAi^	miR-276a knockdown line
BS59897	UAS-mir-276a^OE^	mir-276a Overexpression Line
BS32573	UAS-nej^OE^	nej overexpression line
BS32577	UAS-nej^RNAi^	nej knockdown line

**Table 14:** Genotypes by group.

Group	Abbreviation
Control group with normal gene expression	Hand-Gal4 > W^1118^
Group with heart-specific *nej* gene overexpression	Hand-Gal4 > nej^OE^
Group with heart-specific *nej* gene knockdown	Hand-Gal4 > nej^RNAi^
Group with heart-specific *miR-276a* knockdown	Hand-Gal4 > UAS-miR-276a^RNAi^
Group with Overexpression of Heart-Specific *miR-276a*	Hand-Gal4 > UAS-miR-276a^OE^
Normal Gene Expression Exercise Group	Hand-Gal4 > W^1118^ + E
Exercise Group with Heart-Specific *miR-276a* Knockdown	Hand-Gal4 > UAS-miR-276a^RNAi^ + E
Heart-Specific *miR-276a* Overexpression Exercise Group	Hand-Gal4 > UAS-miR-276a^OE^ + E
Heart-Specific *miR-276a* Overexpression Exercise Group	Cg-Gal4 > W^1118^
Group with adipose tissue-specific overexpression of *miR-276a*	Cg-Gal4 > UAS-miR-276a^OE^
Group with fat tissue-specific miR-276a knockdown	Cg-Gal4 > UAS-miR-276a^RNAi^
Wild-Type Quiet Group	W^1118^-N
Wild-Type Movement Group	W^1118^-E
Normal Gene Expression Exercise Group	Cg-Gal4 > W^1118^ + E
Group with adipose tissue-specific overexpression of *miR-276a*	Cg-Gal4 > UAS-miR-276a^OE^ + E
Exercise group with fat tissue-specific *miR-276a* knockdown	Cg-Gal4 > UAS-miR-276a^RNAi^ + E

**Table 15:** Rearing and intervention environmental conditions.

Condition	Setting
Temperature	25 °C
Humidity	60–70%
Circadian Rhythm	12 h (12 h of daylight, 12 h of darkness)
Stocking Density	30 per tube
Age at Maturity	3 days after emergence

**Table 16:** Primer sequences.

*U6* F	TGGCCCCTGCGCAAGGATG
*miR-276a* F	ctCAGTCAGACCCCATGTAGGAACTTC
*rp49* F	CTAAGCTGTCGCACAAATGG
*rp49* R	AACTTCTTGAATCCGGTGGG
*nej* F	CATCTCATGGGTGCCTCTGG
*nej* R	ACTGTTGAGTTGAGTCATTTGC

## Data Availability

The original contributions presented in this study are included in the article material. Further inquiries can be directed to the corresponding author(s).
